# Using the Veil of Ignorance to align AI systems with principles of justice

**DOI:** 10.1073/pnas.2213709120

**Published:** 2023-04-24

**Authors:** Laura Weidinger, Kevin R. McKee, Richard Everett, Saffron Huang, Tina O. Zhu, Martin J. Chadwick, Christopher Summerfield, Iason Gabriel

**Affiliations:** ^a^DeepMind, London N1C 4DN, United Kingdom; ^b^Department of Psychology, University of Oxford, Oxford OX2 6GG, United Kingdom

**Keywords:** artificial intelligence, ethics, decision-making, fairness, policymaking

## Abstract

The philosopher John Rawls proposed the Veil of Ignorance (VoI) as a thought experiment to identify fair principles for governing a society. Here, we apply the VoI to an important governance domain: artificial intelligence (AI). In five incentive-compatible studies (***N*** = 2, 508), including two preregistered protocols, participants choose principles to govern an Artificial Intelligence (AI) assistant from behind the veil: that is, without knowledge of their own relative position in the group. Compared to participants who have this information, we find a consistent preference for a principle that instructs the AI assistant to prioritize the worst-off. Neither risk attitudes nor political preferences adequately explain these choices. Instead, they appear to be driven by elevated concerns about fairness: Without prompting, participants who reason behind the VoI more frequently explain their choice in terms of fairness, compared to those in the Control condition. Moreover, we find initial support for the ability of the VoI to elicit more robust preferences: In the studies presented here, the VoI increases the likelihood of participants continuing to endorse their initial choice in a subsequent round where they know how they will be affected by the AI intervention and have a self-interested motivation to change their mind. These results emerge in both a descriptive and an immersive game. Our findings suggest that the VoI may be a suitable mechanism for selecting distributive principles to govern AI.

## Significance

The growing integration of Artificial Intelligence (AI) into society raises a critical question: How can principles be fairly selected to govern these systems? Across five studies, with a total of 2,508 participants, we use the Veil of Ignorance to select principles to align AI systems. Compared to participants who know their position, participants behind the veil more frequently choose, and endorse upon reflection, principles for AI that prioritize the worst-off. This pattern is driven by increased consideration of fairness, rather than by political orientation or attitudes to risk. Our findings suggest that the Veil of Ignorance may be a suitable process for selecting principles to govern real-world applications of AI.

The growing integration of Artificial Intelligence (AI) into everyday life raises a critical question: How should society select the principles that govern the deployment and use of AI systems? The need to align AI systems with human morality and values is already salient for applications such as automated vehicles (1), online content recommender systems (2, 3), and social robots (4). It also extends to more powerful types of AI that are expected to take on increasingly important economic and social functions in the future (5–7).

Existing efforts to answer the question of principle selection can largely be categorized into two classes. One set of approaches is morally “intuitionist” in character. These methods aim to capture the moral intuitions that people, including both laypersons and experts, have about AI in order to help guide the development of this technology (8). For example, the Moral Machine experiment (9, 10) leveraged large online surveys to gather data about hypothetical moral dilemmas involving autonomous vehicles, with the ultimate goal of using this information to inform such vehicles’ decision-making. Meanwhile, expert-led processes, such as the Future of Life Institute’s 23 “Asilomar AI Principles” (11) and Atomium-EISMD’s “AI4People” framework (12), foreground the moral intuitions of experts, by proposing sets of principles or values for AI that their authors believe should be respected. The second set of approaches, "theory-led" approaches, proceed differently, starting with a preferred moral theory (such as utilitarianism or virtue ethics) and then reflectively mapping out the implications of that theory for AI. By proceeding in this way, exponents of these specific philosophical positions are able to provide a clearer characterization of what it would mean for AI to be “sufficiently virtuous” or to “promote the greatest good” (13–15).

Although both classes of approach provide novel insights, they also suffer from certain limitations. On the one hand, moral intuitions about technology may conflict with one another, leading to trade-offs or so-called “hard choices” (16, 17) about how to proceed. Moreover, this approach risks capturing preferences that are highly contingent or morally questionable as, for example, when they are influenced by undue personal bias (18). On the other hand, the philosophical expertise required for moral theory-led approaches presents tensions with participatory values and risks unacceptable forms of value imposition when applied to technologies that operate at the societal level (19, 20).

**Fig. 1.**
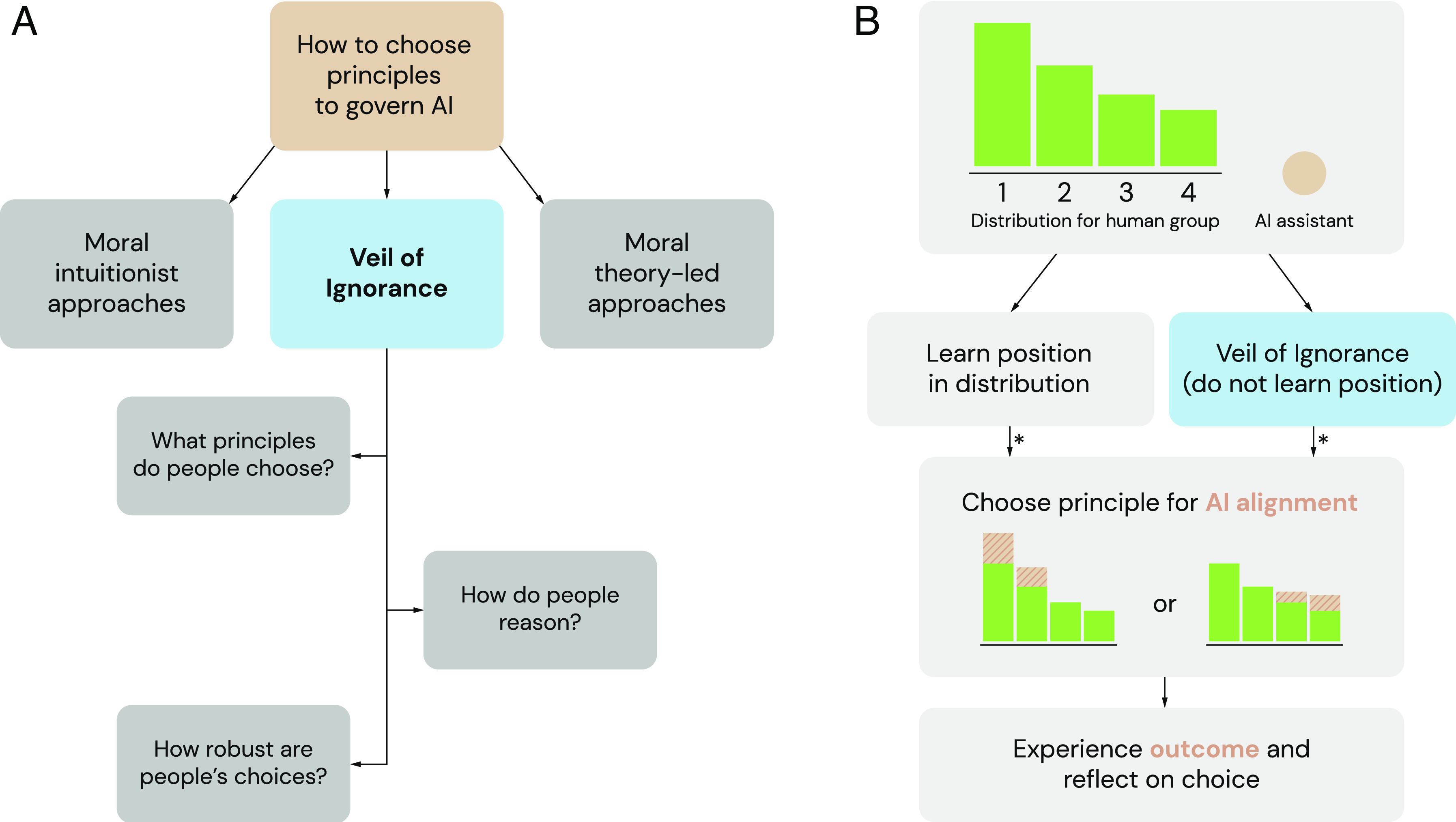
The Veil of Ignorance (VoI) as a possible framework for selecting principles to govern AI systems. (*A*) As an alternative to moral intuitionist and moral theory-led frameworks, we explore the VoI as a fair process for selecting principles to govern AI. (*B*) The VoI can be applied to select principles for AI alignment in distributional situations. A group faces a baseline distribution of resources, with individuals’ positions varying in advantage (here labeled from 1 to 4). The group is set to receive potential assistance from an AI system (here labeled an “AI assistant”). Typically, a decision maker with knowledge of their position in the group selects a principle to guide the assistant. Alternatively, behind the VoI, the decision maker chooses a principle without knowing their position. Once a principle has been selected, the AI assistant enacts the principle and augments the resource distribution accordingly. Asterisks (*) indicate the potential point at which fairness-based reasoning influences judgment and decision-making.

Moreover, while any particular moral theory may be popular among its adherents, there is no guarantee that it possesses widespread support across people with different belief systems (21–23). Given the far-reaching effects of these technologies on people’s lives (24), it is not desirable for AI developers to simply encode some values over others based on their own personal preferences or moral beliefs (25). Instead, the differences in values, interests, and viewpoints that exist in a pluralist society point toward the need for a fair process that can help identify appropriate principles for AI on a society-wide basis (Fig. 1*A*).

Against this backdrop, a third approach aims to identify fair principles to govern AI by drawing upon the Veil of Ignorance (VoI; 20). Originally developed by John Rawls (26), the VoI has become a foundational thought experiment in political philosophy. Building upon the social contract tradition, it asks individuals to select principles of justice for a society without knowing potentially biasing information about the position they will occupy in that society. Ignorance of one’s own personal circumstances, or the personal circumstances of others, forecloses the possibility of prejudicial or self-interested reasoning. Indeed, given that people “do not know how the various alternatives will affect their own particular case [...] they are obliged to evaluate principles solely on the basis of general considerations” and thus to deliberate impartially when selecting principles for society as a whole (26, p. 136–137]. Because no one is unfairly advantaged by this selection mechanism, the resulting principle choice is widely held to be fair.^*^

Drawing upon this framework, Gabriel (20) proposes using the VoI to select principles to govern AI, rather than looking at the impact of the mechanism on case-by-case choices. One advantage of focusing on principles is that they can be described in terms that are easier to understand than complex datasets containing large numbers of case-specific choices. As a result, principles are more easily subjected to public evaluation, debate and endorsement (29–31). Principles also tend to integrate different values into an actionable scheme, thereby avoiding the problem of conflicting values or datapoints that require further trade-offs.

In this paper, we apply the VoI to the challenge of AI value alignment (5, 6) by asking participants to choose a principle for an AI assistant from behind a VoI in an incentive-compatible experimental setting. Participants make their choice before they learn how they will individually be affected. We compare this group to a second group of participants who choose principles to govern AI with full knowledge of their relative position in the group.

In his original proposal, Rawls made a series of predictions about people’s choices that have subsequently become a major topic of interest for political scientists, economists, and social psychologists (32–41). For example, Rawls argued that people would adopt a maximin strategy when choosing principles of justice behind the VoI, and hence, they would endorse principles that prioritize the worst-off.^†^ In contrast, John Harsanyi made a different prediction: People would choose principles that maximize overall return behind the VoI (42). Rawls also held that people would reach a state he calls “reflective equilibrium,” where they would continue to endorse the conclusions generated by reasoning behind the VoI even after the veil was lifted (43, p. 17).

Previous empirical studies on the VoI primarily focus on selecting principles to govern the state or the distribution of wealth in society (32, 35, 44).^‡^ In laboratory studies, the VoI tends to promote greater prioritization of the worst-off (35, 36, 40, 47), though Frohlich et al. (32, 33) found support for “hybrid” principles that combine a welfare floor with elements of maximization. With regard to the kind of reasoning people employ, some studies suggest that the VoI is primarily a concept about risk (35, 44), whereas others identify prosocial preferences as the key driver of participant behavior. For example, Schildberg-Hörisch (40) observed that participants behind the VoI assigned greater priority to the worst-off when this decision affected other people, compared to when it only affected themselves.

Building on these findings, the present work attempts to directly test Rawls’ propositions about the VoI in the domain of AI alignment. We investigate whether individuals who reason behind the VoI more often choose to prioritize the worst-off, and whether they are more likely to endorse their choices upon reflection after the veil is lifted, in an effect we term ‘reflective endorsement’. Finally, we investigate what factors influence reasoning behind the VoI by eliciting participant explanations for why they made their choices and by measuring participant attitudes toward risk and political preferences. Drawing upon recent findings in the field of experimental psychology (34, 41, 48), we hypothesize that the VoI is more than a mechanism to elicit political preferences or attitudes to risk, and that unprompted consideration of fairness behind the VoI—what we call “fairness-based reasoning”—plays a relatively greater role influencing choice, compared to a Control condition. We test these effects both in a descriptive task and in an immersive, real-time harvesting game. If the VoI leads to greater fairness-based reasoning and elicits preferences that are endorsed upon reflection when applied to AI, then these preferences have the potential to serve as a suitable focal point for the creation of aligned AI systems.

## Experiment Design and Results

We recruited *N* = 2,508 participants (after exclusion: 2,101) from Prolific (49) across five online studies (median age range: 35 to 44; gender: 57.7% female, 40.6% male, 0.8% trans, nonbinary, genderqueer, or genderfluid). Participants completed an incentivized computer-based harvesting task, each in a group with three ostensible humans (actually computer bots) and an AI assistant. The goal in the task for participants was to “harvest trees,” potentially with support from the AI assistant. Participants completed either a descriptive version of this task (with no real-time component to harvesting trees; studies 1, 2, 4, and 5) or an immersive version (wherein participants harvested trees by controlling an avatar in a real-time, virtual environment; study 3).

In each experiment, before the task began, participants were randomly assigned to one of four possible positions within the group. The positions varied in terms of harvesting productivity, ranging from a severely disadvantaged position (i.e., a low expected harvest) to the most advantaged position (i.e., a high expected harvest; see *Methods* for detailed designs). In each study, participants were asked to choose between two possible principles to govern the AI’s behavior. The principles were a prioritarian principle that assigns priority to the worst off; and a maximization principle that maximizes the return over the entire group, regardless of which group member will benefit.^§^

Each principle was represented with a visual depiction of the harvest outcomes across the group (i.e., a bar chart; Fig. 1*B*), and with a textual label: “Collect trees for the players who are most disadvantaged at the start of the round” (prioritarian principle) and “Collect as many trees as possible” (maximization principle), respectively. An AI assistant following the prioritarian principle would support participants in the comparatively disadvantaged positions. By contrast, as the advantaged positions offered greater harvests, an AI assistant following the maximization principle would support participants in those positions. Participants made the choice between principles either without knowing which position they would be assigned to, and thus without knowing which principle would benefit them (VoI condition); or with full knowledge of their position and of how they would be affected (Control condition).

After conducting an initial study with this basic protocol (study 1, *N* = 239), we expanded our investigation with a high-powered, preregistered study (study 2, *N* = 890). We then conducted another high-powered, preregistered study in an immersive setting (study 3, *N* = 891). This protocol placed participants in a real-time, interactive version of the harvesting game, in line with methods used in contemporary AI research (50, 51). Finally, we ran two additional study variations to test the robustness of any effects behind the VoI. One examined the role of prosociality in the decision-making effects of the VoI (40) by explicitly informing participants that the other individuals in their group were computerized bots, rather than other human participants (study 4, *N* = 253). The last tested the importance of linguistic representations of the principles (52) by replacing the verbal descriptions of the principles with the content-agnostic labels “Principle A” and “Principle B” (study 5, *N* = 235).

**Fig. 2.**
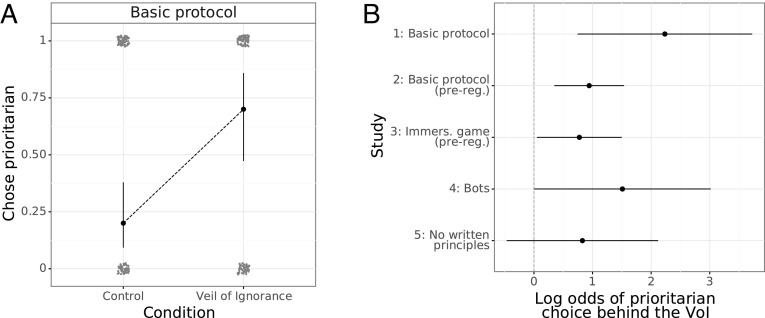
Effect of the Veil of Ignorance (VoI) on the likelihood of choosing the prioritarian principle to govern AI behavior. Error bars represent 95% confidence intervals. (*A*) With the basic protocol (study 1), the VoI exerted a significant effect on participant decision-making, increasing the likelihood of participants choosing the prioritarian principle, *P* = .003 (logistic regression). (*B*) Log odds from logistic regressions indicate similar effects of the VoI on decision-making in study 2, *P* = .002, study 3, *P* = .032, and study 4, *P* = .049, but not in study 5, *P* = .21.

The VoI significantly increased the likelihood of participants choosing the prioritarian principle over the maximization principle in study 1, OR = 9.3, 95% CI [2.7, 37.6], *p* = .003 (logistic regression; Fig. 2*A*). This effect replicated in study 2, OR = 2.6, 95% CI [1.5, 4.5], *P* = .002; study 3, OR = 2.2, 95% CI [1.2, 4.0], *P* = .036; and study 4, OR = 4.5, 95% CI [1.4, 16.8], *P* = .049 (Fig. 2*B*). Surprisingly, even though participants in study 4 were aware that they were playing the game alongside bots (and not other human participants), those behind the VoI were still more likely to choose the prioritarian principle than those in the Control condition. In contrast, we find a boundary condition for the effect of the VoI in study 5, *P* = .21 (*SI Appendix*, Table S1).

We operationalize Rawls’ proposition about reflective equilibrium in a “reflective endorsement” test whereby participants, having completed the harvest task—and thus having experienced the effect of their chosen principle—are randomly assigned to a new position in the group and asked whether they would endorse the same principle again. With full information about how they would be affected, do participants continue to endorse their original principle choice?

We were particularly interested in the participants who faced a self-interested motivation to change: that is, the subset of participants who had received assistance based on their chosen principle during the previous round but would be excluded from assistance by the same principle on the basis of their new position. Two groups faced a motivation to change. First, participants who chose the prioritarian principle and occupied a disadvantaged position in the original task but who were assigned to an advantaged position in the new ordering, experienced an incentive to choose the maximization principle. Second, participants who originally chose the maximization principle and had occupied an advantaged position but who were subsequently assigned to a disadvantaged position, faced a motivation to choose the prioritarian principle.

**Fig. 3.**
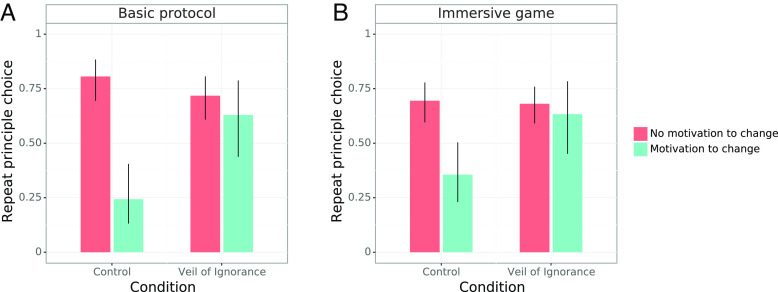
The Veil of Ignorance (VoI) increased the likelihood of participants maintaining their principle choices (reflective endorsement), specifically among those facing a self-interested motivation to change their choice. Error bars reflect 95% confidence intervals. (*A*) Reasoning behind the VoI increased the likelihood of participants maintaining their principle choice, specifically if they faced a motivation to change their choice, *P* = .005 (study 1; logistic regression). (*B*) Similarly, participants who experienced a motivation to change were more likely to maintain their principle choice if they had chosen behind the VoI, *P* = .036 (study 3; logistic regression).

Participants in the VoI condition who encountered the motivation to change were more likely to repeat (i.e., endorse) their previous choice, compared to participants who faced such a motivation in the Control condition, in both the basic protocol and the immersive game study 1, OR = 8.6, 95% CI [2.3, 34.0], *P* = .005; study 2, OR = 3.8, 95% CI [1.9, 7.7], *P* <  .001; study 3, OR = 3.3, 95% CI [1.1, 10.5], *P* = .036 (logistic regressions; Fig. 3 and *SI Appendix*, Table S2). We observed no difference in reflective endorsement between the VoI and Control condition among participants who faced a motivation to change in study 4 (*P* = .21) and study 5 (*P* = .10).

We investigated whether the two principles were equally likely to be endorsed upon reflection. In study 2, 66% of participants in the VoI group and 30% of participants in the Control condition repeated their choice of the prioritarian principle, despite facing a motivation to adopt the maximization principle. In contrast, of those who had chosen the maximization principle and faced a new motivation to adopt the prioritarian principle, only 29% in the VoI group and 33% in the Control condition repeated their choice. Overall, the tendency for VoI participants to repeat their choice when facing a motivation to change was significantly pronounced for participants who initially chose the prioritarian principle, *χ*^2^ = 9.56, *P* = .002 (Breslow-Day test; *N*_analysis_ = 262). That is, participants behind the VoI were more likely to reflectively endorse an initial prioritarian choice than an initial maximization choice.^¶^

We next tested what factors influence decision-making behind the VoI, as measured through several questionnaire instruments after the task: attitudes to risk (54), political preferences (55, 56), and the frequency with which participants made unprompted references to fairness considerations when explaining their choice, (*Methods*, (57)). Attitudes to risk predicted principle choice behind the VoI with *R*^2^ = 6.5% in study 1 (logistic regression; *SI Appendix*, Table S5). Liberal-conservative political orientation predicted principle choice behind the VoI with *R*^2^ = 2.5%, and left–right political orientation was predictive of principle choice with *R*^2^ = 1.0%. In contrast, fairness considerations explained a greater proportion of variability in principle choice, with *R*^2^ = 51.2%. These patterns extended to studies 2, 3, 4, and 5 (*SI Appendix*, Table S5). Surprisingly, invocation of fairness considerations predicted choices even when participants knew they played alongside bots in study 4, *R*^2^ = 29.8%, whereas risk preferences predicted principle choice with only *R*^2^ = 8.9%. Participants’ justifications for their principle choice frequently made clear the connection between the VoI and their concerns for fairness. As one participant wrote, “Since I did not know whether I would be allocated to a field with more or less trees when making the decision, I figured I might as well make it more fair for everyone.”

**Fig. 4.**
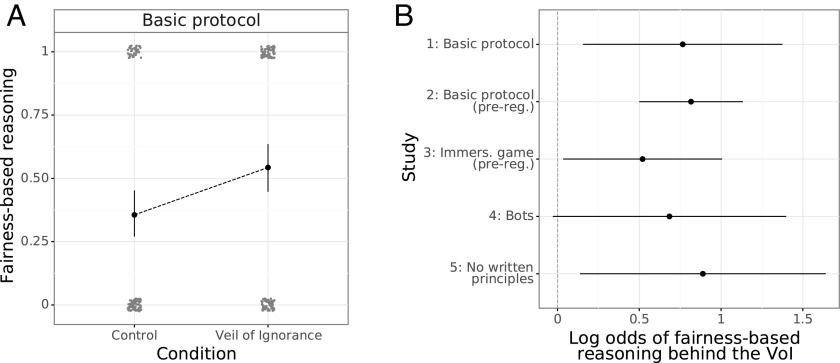
Participant invocation of fairness-, equality-, and equitability-related concepts in explanations of their principle choices. Error bars represent 95% confidence intervals. (*A*) In study 1, participants who selected a principle behind the Veil of Ignorance (VoI) were significantly more likely to explain their decision in terms of fairness than participants who selected with knowledge of their personal position in the group, *p* = .014 (logistic regression). (*B*) After adjusting for multiple comparisons, log odds from logistic regressions indicate similar effects of the VoI on decision-making in study 2, *P* <  .001, study 3, *P* = .036, and study 5, *P* = .020, but not study 4, *P* = .06.

Given the apparent role of fairness-based reasoning in preference for prioritarian choice, we sought to understand whether this primarily emerged behind the VoI or was prompted equally regardless of participants’ awareness of their position. Participants engaged in fairness-based reasoning to a significantly greater degree in the VoI condition than in the Control condition, across all studies: study 1, OR = 2.2, 95% CI [1.2, 3.8], *P* = .014; study 2, OR = 2.3, 95% CI [1.7, 3.1], *P* <  .001; study 3, OR = 1.7, 95% CI [1.1, 2.7], *P* = .036; and study 5, OR = 2.4, 95% CI [1.3, 4.7], *P* = .020 (logistic regressions; Fig. 4). After correcting for multiple comparisons, we observe no difference in fairness-based reasoning between conditions in study 4, *P* = .06. As expected, participants behind the VoI who were told that they were playing alongside humans (study 2) more often invoked fairness-based reasoning than those behind the VoI playing alongside bots (study 4), OR = 1.7, 95% CI [1.1, 2.6], *P* = .023 (logistic regression).

Finally, to explore whether fairness-based reasoning is a factor leading to increased reflective endorsement, we tested its predictiveness for decisions after the veil is lifted. Participants who invoked fairness in their reasoning process behind the VoI tended to settle on a more stable principle choice that they endorsed upon reflection: fairness-based reasoning increased the likelihood of VoI participants to reflectively endorse their choice, despite facing a motivation to change, in study 1, OR = 7.6, 95% CI [1.4, 51.2], *P* = 0.024 (*N*_analysis_ = 27). We observed the same effect in study 2, OR = 4.8, 95% CI [2.1, 11.8], *P* = .001 (*N*_analysis_ = 96), and study 3, OR = 3.3, 95% CI [1.3, 9.0], *P* = .036 (*N*_analysis_ = 78).

## Discussion

How do people choose principles for governing an AI assistant when reasoning from behind the VoI? In our studies, we observe three effects. First, participants behind the VoI more often choose principles favoring the worst-off, relative to those in the Control condition. Second, converging with Rawls’s idea of a “reflective equilibrium,” participants who experienced the VoI more often endorse their choice upon reflection than participants in a Control condition. Third, when explaining their reasoning, participants in the VoI condition more often make unprompted reference to fairness-based concepts. Notably, each of these three effects holds in a descriptive game (studies 1 and 2) as well as in a fully immersive game (study 3).

The idiosyncratic characteristics of modern AI systems distinguish the domain of AI alignment from prior domains in which the VoI has been studied.^#^ Nonetheless, we find that participants in the VoI condition prioritize the worst-off, convergent with results from other areas of inquiry, e.g., wealth distribution; (32, 40). This effect holds in all studies bar one where the distributional representations of the principles were accompanied by the labels “Principle A” and “Principle B” (study 5), rather than linguistic descriptions, highlighting the importance of a rich representation of the principles. Linguistic relativity theories propose that language influences the way that people think (59), potentially including the way they contemplate and understand moral principles (52). Our findings support the idea that moral cognition, “like all ‘higher psychological functioning’ [...] is necessarily mediated by words, language, and forms of discourse” (60, p. 355]. Huang et al. (41, p. 23993] suggest that the VoI “requires a kind of spontaneous ‘microphilosophizing’ to produce its effect”. Building upon this insight, our results suggest that absent general linguistic descriptions of principles, participants may be less likely to engage in effectual moral reasoning as compared with situations where principles are explained in such terms.

Relatedly, the kind of reasoning that drives choices behind the VoI has long been the subject of research. Previous empirical explanations of prioritarian preferences behind the VoI center largely on risk aversion: To avoid the worst possible outcome to themselves, participants select the prioritarian choice (35, 44). However, we find that fairness-based considerations are a stronger driver of participant decisions. In all studies, participants behind the VoI more often make unprompted reference to fairness when explaining their reasoning process, compared to those in the Control group. Notably, this finding held even when participants were provided with distributional representations, but no linguistic description of each principle (study 5). This finding suggests that, rather than being solely about risk, the VoI serves as a mechanism to elicit fairness-based reflection and decision-making.

In an empirical test of a “reflective endorsement” effect, we further find evidence for Rawls’s proposition that choices made behind the VoI are more likely to be endorsed even after the veil is lifted. The VoI triggers reasoning about fairness, which in turn leads participants to prioritize the worst off. This chain of reasoning more often induces choice “stickiness” in the VoI condition: After the veil is lifted, participants continue to endorse their choices despite facing a self-interested motivation to change. Taken together, this causes prioritarian principle choices to be “stickier” than maximization principle choices, with participants more often endorsing their decision upon reflection. In studies 4 (where participants knew the other members of their group were computer bots) and 5 (where participants are presented only with visual principle descriptions), this chain of reasoning was attenuated: The VoI manipulation proves insufficient to motivate prioritarian preferences (study 5) or to motivate participants to feel sufficiently committed to “stick” to their choices (study 4).

What do these findings tell us about the selection of principles for AI in the real world? First, the effects we observe suggest that—even though the VoI was initially proposed as a mechanism to identify principles of justice to govern society—it can be meaningfully applied to the selection of governance principles for AI. Previous studies applied the VoI to the state, such that our results provide an extension of prior findings to the domain of AI. Second, the VoI mechanism demonstrates many of the qualities that we want from a real-world alignment procedure: It is an impartial process that recruits fairness-based reasoning rather than self-serving preferences. It also leads to choices that people continue to endorse across different contexts even where they face a self-interested motivation to change their mind. This is both functionally valuable in that aligning AI to stable preferences requires less frequent updating as preferences change, and morally significant, insofar as we judge stable reflectively endorsed preferences to be more authoritative than their nonreflectively endorsed counterparts. Third, neither principle choice nor subsequent endorsement appear to be particularly affected by political affiliation—indicating that the VoI may be a mechanism to reach agreement even between people with different political beliefs. Lastly, these findings provide some guidance about what the content of principles for AI, selected from behind a VoI, may look like: When situated behind the VoI, the majority of participants instructed the AI assistant to help those who were least advantaged.

The landscape of AI applications and services is both complex and multifaceted. In order to cast further light on the potential for the VoI as an alignment procedure, and to understand how well these findings map onto other domains, future research may need to explore how the mechanism functions across a wider range of realistic AI applications and contexts. This includes exploring different domains of application (e.g., healthcare, education, manufacturing, etc.) and exploring scenarios that contain additional factors that could influence participant choices, such as whether the AI system is ostensibly “owned” by one of the parties to begin with refs. 61–64, or what overall level of capability the AI system possesses (65–67).

To further understand the nature of the preferences elicited behind the VoI, it would also be useful to present participants with a larger set of principles from which to choose. In particular, it may be worth testing the appeal of certain kinds of “hybrid” principles for AI, combining a prioritarian-like guaranteed return to the worst-off with constrained maximization, given support for these variants in other experimental settings (32, 33, 37). Equality-inspired principles, such as “provide equal benefits to everyone” and “spend equal time assisting everyone”, also merit investigation (68). Finally, given the anticipated global impact of AI, future research should examine cross-cultural differences between preferences to govern AI (25). It is possible that different demographic groups endorse different principles for AI from behind the VoI and that there is variation at local, regional, or global levels. Encouragingly, other applications of the VoI exhibit considerable cross-cultural stability (35, 36, 38, 39).

A key challenge for society is to determine the values and principles with which to align AI systems. Underlying this is both a moral and a political challenge: Which alignment procedure is fair, robust, and scalable? Because this is a normative debate, empirical evidence such as the results presented in this paper cannot conclusively settle the question. However, they can inform the answer (69). Here, we show that the VoI is one viable option for selecting principles for AI alignment, with the particular benefits that it recruits fairness-based reasoning—pushing participants to reflect and think about their choices—and results in the elicitation of preferences that people endorse in the face of a self-interested motivation to change. Moreover, the VoI has already been proposed, with some success, as a solution to normative questions in other real-world domains such as tax policy (47, 70). The VoI, therefore, allows us to identify principles that are promising candidates for consideration in the context of ongoing discussion about how best to align AI with human values.

## Materials and Methods

All studies were approved upon independent review by the Human Behavioural Research Ethics Committee at DeepMind (#19/002). We collected informed consent from all participants before their study sessions began.

The data and code necessary for reproducing all analyses and figures are available at https://osf.io/eapqu/.

### Study 1.

#### Sample.

We recruited an online sample through Prolific (*N* = 239, median age range: 35 to 44). Inclusion criteria for recruitment were residence in the United Kingdom, fluency in English language, and completion of at least 200 previous studies with an approval rate of 95% or more. Participants were excluded from analysis if they did not answer both questions in the comprehension test correctly (described further in the study procedures). Approximately 56.1% of recruited participants identified as female, 42.2% as male, and 0.8% as nonbinary.

#### Procedure.

The study was implemented using a custom-built platform that combines standard questionnaire functionality with the ability to run games for both human and AI players.

The study followed a between-participant design. Participants were randomly assigned to either the Veil of Ignorance (VoI) condition or the Control condition.

In both conditions, participants were told that the harvesting task involved a group of four human group members and one AI assistant (referred to simply as “an AI” for participants). In actuality, the other three “human” group members were computer-controlled bots.^||^ The goal for each group member was to gain as large a harvest as possible from their individual territory (“field”); participants received a bonus that increased for each tree in their harvest. The AI assistant also contributed to the tree harvest. It increased the harvest for different group members as a function of the “principle” guiding its behavior. The instructions were explicit that participants’ overall harvest (i.e., the human and AI harvest in their field combined) would determine their bonus (full instructions in *SI Appendix*). The task involved four fields varying in tree density. Before the task began, each participant was randomly assigned to one of the fields, resulting in different levels of advantage for harvesting productivity. Participants were told that they had been randomly selected out of their group to choose a principle to govern the AI assistant’s behavior, thus determining whom the AI assistant would support. Participants were instructed that no other group member would at any point find out that they were the “decision-maker,” so their choice would be anonymous.

Participants then learned about the two principles that the AI assistant could follow. One principle corresponded to maximizing the overall harvest of the group (a maximize principle). The other principle corresponded to maximizing the minimum outcome for the group—that is, helping the worst-off group member (a prioritarian principle). The principles were described as “Collect as many trees as possible” and “Collect trees for the players who are most disadvantaged at the start of the round,” respectively. Participants responded to two comprehension questions to assess whether they had correctly learned how the principles mapped to example distributions of AI assistance between the fields. The comprehension test was incentivized with a bonus of £0.25 per question.

After completing the comprehension test, participants in the Control condition were informed of their position for the upcoming round of harvesting. Participants in both conditions then chose which principle the AI assistant should follow in this round. Participants in the VoI condition were informed of their position only after submitting their choice of principle.

The study then presented participants with a bar chart depicting the outcome of the harvesting round (i.e., a visual representation of the group’s harvest outcomes). The bar chart showed each group member’s harvest and the AI contribution to each position (Fig. 1*B*). A blue arrow indicated the participant’s own harvest.^**^

After the harvesting round, the study prompted participants with the open-ended question “Why did you choose this principle?” In both conditions, the study then selected a random field for each participant and asked which principle they would choose in an additional round with that position. Participants indicated their choice for the additional (hypothetical) round.

The study concluded with a short questionnaire. The questionnaire solicited risk preferences (44, 54), political orientation between liberal-conservative and between left–right (55, 56), and collected demographic information including age range and gender identity. Finally, participants were debriefed on the study purpose and the use of deception. To ensure no disadvantage to participants who had forsaken reward to benefit other human group members, participants were paid the maximum harvesting bonus (£1.50) irrespective of their choices, in addition to any bonus earned from the comprehension test. On average, participants completed the study in 11.4 min.

#### Analysis.

Thirty participants answered at least one comprehension question incorrectly and were excluded from analysis. Unless otherwise noted, each analysis included the remainder of the sample (*N*_analysis_ = 209). We adjust for multiple comparisons with the Hochberg procedure before reporting *P* values (71) and confidence intervals (72). Unadjusted statistics are reported in *SI Appendix*.

To test the effect of the VoI on principle choice, we fit a logistic regression predicting whether participants chose the prioritarian principle, using two inputs: condition and the interaction between condition and position. The interaction term accounts for the effect of position on principle choice in the Control condition.

To test the effect of the VoI on reflective endorsement, we first coded a dummy variable indicating whether participants faced the “motivation to change” their choice in a new round of the harvest task. Participants were identified as facing the motivation to change if

- they had previously chosen the prioritarian principle and occupied the low-density fields, but in the additional round were assigned a high-density field (“motivation to maximize”)
- or they had previously chosen the maximize principle and occupied the high-density fields, and in the new round were assigned a low-density field (“motivation to prioritize”).

In both hypothetical situations, selecting the original choice for a second time would sacrifice the maximum bonus. Participants who chose the same principle in the additional round as in the main round were taken to “reflectively endorse” their previous choice, despite the motivation to change. For participants who did not face the motivation to change, repeating their prior choice could be driven by either reflective endorsement or the goal of maximizing the bonus payment.

We then fit a logistic regression predicting whether participants repeated their original principle choice, using three inputs: condition, the presence (or absence) of a motivation to change, and the interaction between condition and the motivation to change.

Fairness-based reasoning was measured from participants’ open-ended explanations to the question asking why they chose the selected principle. Two annotators coded responses on whether or not they invoked notions of “fairness, equality, or equitability”. Agreement between the two raters was high, as measured by Krippendorff’s alpha (*α* = 0.91). A third rater coded any responses where the first two raters disagreed. All raters were blind with regard to participant condition and did not observe any additional information about the participants during the coding process. Responses that were marked by at least two raters to invoke a notion of fairness, equality, or equitability were recorded as indicating fairness-based reasoning.

To assess the relationship between risk attitudes, political preferences, and fairness-based reasoning with decision-making behind the VoI, we fit simple logistic models regressing prioritarian choice on each variable for participant decisions in the VoI condition. We computed Nagelkerke’s *R*^2^ to compare goodness-of-fit between the models (73).

We assess the relationship between fairness-based reasoning and the VoI by fitting a simple logistic model regressing fairness-based reasoning on condition. To evaluate the role of fairness-based reasoning on participants’ responses to the motivation to change behind the VoI, we fit a logistic model regressing reflective endorsement on fairness-based reasoning, specifically among participants who experienced a motivation to change in the VoI condition.

### Study 2.

#### Sample.

We recruited an online sample through Prolific (*N* = 890, median age range: 35 to 44). The study applied the same inclusion and exclusion criteria as study 1. Approximately 60.2% of recruited participants identified as female, 38.9% as male, and 0.3% as nonbinary or genderqueer.

#### Procedure.

The study was preregistered (protocol available at https://osf.io/cfvm3). It was implemented using the same custom-built platform as study 1 and followed the same protocol as study 1. We recruited a larger sample than originally preregistered due to an update in our informal power analysis for the motivation-to-change test. On average, participants completed the study in 11.3 min.

#### Analysis.

A total of 114 participants answered at least one comprehension question incorrectly and were excluded from analysis. Unless otherwise noted, each analysis included the remainder of the sample (*N*_analysis_ = 776). We adjust for multiple comparisons with the Hochberg procedure before reporting *P* values and confidence intervals. Unadjusted statistics are reported in *SI Appendix*.

Analysis generally followed the same plan as study 1. We include one additional analysis, due to the larger number of participants who experienced the motivation to change their principle choice in the hypothetical round relative to study 1 (as a result of the higher study power). We conduct a Breslow-Day test to assess whether the association between condition and reflective endorsement differs by the specific type of motivation to change (motivation to maximize or motivation to prioritize). As in study 1, there was high agreement between the first two raters on participants’ use of fairness-based reasoning (*α* = 0.89).

### Study 3.

#### Sample.

We recruited an online sample through Prolific (*N* = 891, median age range: 35 to 44). The study applied the same inclusion and exclusion criteria as studies 1 and 2. Approximately 60.8% of recruited participants identified as female, 36.9% as male, and 1.2% as trans, nonbinary, genderqueer, or genderfluid.

#### Procedure.

The study was preregistered (protocol available at https://osf.io/45rqw). It was implemented using the same custom-built platform as studies 1 and 2. It followed the same basic protocol, with one broad change: It replaced the descriptive harvesting task with an immersive harvesting game implemented in DeepMind Lab2D (74). To acquaint participants with the game controls, the study included a “tutorial” round before introducing the AI assistant and principle choices.

In the immersive harvesting game, participants controlled an avatar (blue figure) using their keyboard (*SI Appendix*, Fig. S1). They could move around and harvest trees in one field within a 2D gridworld. Participants were told that they would play alongside other human group members (red figures) who would be assigned to other fields. Human group members could not cross the boundaries between fields. Participants learned that they would also play alongside an AI assistant (beige figure) that could cross field boundaries and collect trees in multiple fields. As in the prior studies, the fields varied in tree density; participants were told that harvesting was less efficient in sparser fields. Trees regrew five seconds after being harvested.

When the principles were introduced, participants observed 50-s videos showing examples of the AI assistant following each principle. Participants completed an additional comprehension test asking them to watch several new 50-s videos and match each to the corresponding principle. (The exact behavior of the AI assistant in these videos differed from the behavior in the instructional videos.) Participants were not presented with bar charts of outcomes in the instructions, so their impressions of distributive properties of the principles in these instructions were informed by the videos of AI behavior.

After completing the comprehension test, participants were told that they had been selected to choose a principle for the AI assistant. As in prior studies, participants in the Control condition (but not the VoI condition) were informed of their position for the game. All participants indicated their principle choice. Participants then played the immersive harvesting game for 60 s, randomized between one of two tree layouts (to control for the idiosyncratic properties of any particular layout). The red avatars (ostensibly representing other human group members) followed trajectories recorded from a pilot sample of prior participants, selected to reflect median harvesting capability of this prior sample. The AI assistant followed trajectories that contributed primarily to the two more-advantaged positions (maximization principle) or the two less-advantaged positions (prioritarian principle). The actual AI trajectory for a session was randomly sampled from a set of four for each principle (to control for the idiosyncratic properties of any particular trajectory). After participants completed the game, the study presented a bar chart that depicted the outcomes that they, the AI assistant, and the bots had generated. On average, participants completed the study in 20.7 min.

#### Analysis.

A total of 184 participants answered at least one comprehension question incorrectly and were excluded from analysis. Unless otherwise noted, each analysis included the remainder of the sample (*N*_analysis_ = 707). We adjust for multiple comparisons with the Hochberg procedure before reporting *P* values and confidence intervals. Unadjusted statistics are reported in *SI Appendix*.

We incorporated an additional criterion for the reflective endorsement analysis. In the immersive setting, it was possible for participant behavior to move group harvest levels out of distribution: Depending on their skill or effort level, participants could score more than the position more advantaged than theirs or less than the position less advantaged. This provides an alternative reason not to endorse a prior principle choice. For example, a participant might occupy a high-density field but score the lowest in the group and thus consider themselves the most disadvantaged; but they would not receive help from the AI assistant following the prioritarian principle. Because their relative harvest deviated from the expected distribution, 421 participants were thus excluded specifically from the reflective endorsement analysis. Analysis otherwise followed the same plan as study 1. As in the previous studies, there was high agreement between the first two raters on participants’ use of fairness-based reasoning (*α* = 0.82).

### Study 4.

#### Sample.

We recruited an online sample through Prolific (*N* = 253, median age range: 35 to 44). The study applied the same inclusion and exclusion criteria as studies 1, 2, and 3. Approximately 47.4% of recruited participants identified as female, 50.6% as male, and 1.2% as nonbinary or genderfluid.

#### Procedure.

The study was implemented using the same custom-built platform as studies 1, 2, and 3. It followed the same basic protocol as study 1, with one change: Participants were truthfully informed that the other members of their group were “bots” (rather than being told that they were other human participants). On average, participants completed the study in 12.0 min.

#### Analysis.

Forty participants answered at least one comprehension question incorrectly and were excluded from analysis. Unless otherwise noted, each analysis included the remainder of the sample (*N*_analysis_ = 213). We adjust for multiple comparisons with the Hochberg procedure before reporting *P* values and confidence intervals. Unadjusted statistics are reported in *SI Appendix*. Analysis generally followed the same plan as study 1. We include one additional analysis, comparing the effect of the VoI on fairness-based reasoning between study 1 and study 4. To do so, we fit a joint logistic regression with an additional dummy variable representing the study and subsequently compare estimated marginal means for the two studies. As in the previous studies, there was high agreement between the first two raters on participants’ use of fairness-based reasoning (*α* = 0.90).

### Study 5.

#### Sample.

We recruited an online sample through Prolific (*N* = 235, median age range: 35 to 44). The study applied the same inclusion and exclusion criteria as studies 1, 2, 3, and 4. Approximately 49% of recruited participants identified as female, 48% as male, and 1% as nonbinary.

#### Procedure.

The study was implemented using the same custom-built platform as studies 1, 2, 3, and 4. It followed the same basic protocol as study 1, with one change: The verbal description of each principle was replaced with an abstract label (“Principle A” and “Principle B”). Participants still observed the bar charts visually depicting the outcome distributions for each principle. On average, participants completed the study in 11.5 min.

#### Analysis.

Thirty-nine participants answered at least one comprehension question incorrectly and were excluded from analysis. Unless otherwise noted, each analysis included the remainder of the sample (*N*_analysis_ = 196). We adjust for multiple comparisons with the Hochberg procedure before reporting *P* values and confidence intervals. Unadjusted statistics are reported in *SI Appendix*. Analysis otherwise followed the same plan as study 1. As in the previous studies, there was high agreement between the first two raters on participants’ use of fairness-based reasoning (*α* = 0.90).

## Supplementary Material

Appendix 01 (PDF)

## Data Availability

Preregistered protocols, analysis scripts and anonymized experimental data are available at https://osf.io/eapqu/ (75).

## Footnotes

^*^Notably, the Veil of Ignorance is only one way in which impartial reasoning can be modeled. For wider discussion of varieties of impartiality, see (27, 28).

^†^Rawls refers to this principle as the “difference principle”.

^‡^An exception is Huang et al. (41), who apply the VoI to moral dilemmas (45, 46).

^§^Although the maximization principle bears some resemblance to a utilitarian moral framework, the experimental principle and moral doctrine differ in critical ways. The maximization principle is concerned with resources, whereas the utilitarian position focuses on subjective welfare. As a result, in cases where resources have declining marginal utility, the utilitarian may support principles that disproportionately favor the least well-off.

^¶^Similar descriptive patterns emerge in studies 1 and 3, the two other studies with a significant reflective endorsement effect (*SI Appendix*, Tables S3 and S4). However, the sample sizes they offer (*N*_analysis_ = 64 and *N*_analysis_ = 85, respectively) do not meet the cell counts advised for contingency-table analysis (53).

^#^Previous studies primarily apply the VoI to selecting principles to govern society and specifically the state. AI differs from the state in several substantive ways. For example, the primary role of AI systems at this time is additive, where AI systems augment the productivity or wealth of those who have access to it (58). AI systems do not collect taxes and redistribute wealth, which is a central responsibility for the state. Further, AI systems are frequently designed as single interactive agents, such as voice assistants, rather than as community-facing governance institutions. Finally, although AI is regularly conceived of in physically embodied forms (e.g., a robot helper supporting agricultural harvesting), the state is rarely embodied in this manner.

^||^A single-participant design offered two key advantages for the study. First, it allowed each participant to experience their own principle choice, enabling the test of reflective endorsement for all participants. Second, it reduced the dropout rate since the withdrawal of a single participant for a group study would have corrupted data collection for three other participants. The study maintained the impression of other participants through wait screens of a random duration between 2 to 8 seconds at various points throughout the study (e.g., at the end of the comprehension test).

^**^For full task details and screens as displayed to participants, *SI Appendix*.
