# Supplementary material for: Using the Veil of Ignorance to align AI systems with principles of justice
Source: Proc Natl Acad Sci U S A. 2023 Apr 24;120(18):e2213709120. doi: 10.1073/pnas.2213709120 (PMC10160973; doi:10.1073/pnas.2213709120)
Supplement: Supplementary file 1 — Appendix 01 (PDF) [file pnas.2213709120.sapp.pdf]

Supporting Information for

Using the Veil of Ignorance to align AI systems with principles of justice

Laura Weidinger, Kevin R. McKee, Richard Everett, Saffron Huang, Tina O. Zhu, Martin J. Chadwick, Christopher Summerfield, and Iason Gabriel

Corresponding authors: Laura Weidinger and Kevin R. McKee
E-mail: lweidinger@deepmind.com and kevinrmckee@deepmind.com

This PDF file includes:

Supporting text
Figs. S1 to S20
Tables S1 to S8
SI References

Supporting Information Text

All five studies received a favourable opinion from the Human Behavioural Research Ethics Committee at DeepMind (#19/004).

Task details

Across all studies, we use a colourblind-friendly palette for the bar charts indicating harvesting results for the participant's group. We similarly use a colourblind-friendly palette for the colour of participant and AI avatars in study 3.

Each study unfolded in the same general order. At the beginning of each study, participants were randomised between the VoI and Control conditions. In the basic protocol (studies 1 and 2), participants proceeded through the following stages:

1. Read instructions and rules for the harvesting task, including written descriptions and visual demonstrations of the principles (Figs. S2, S3, and S4);

2. Take comprehension test on the principles (Figs. S5 and S6);

3. Select principle for the AI assistant to follow, complete the harvesting task, and see the resulting group outcome distribution (Figs. S7 and S8);

4. Complete the post-task questionnaire.

In the Control condition, participants were shown their position in the group distribution (Fig. 8a) before they chose a principle for the AI assistant to follow (Fig. 8b-d). Participants in the VoI condition were not informed of their position before they selected a principle. They instead learned this information after principle selection.

After the distributive harvesting task, the study asked participants several questions about their principle choice. First, the study reminded participants which principle they chose. Participants reported their confidence that they "made the right choice" on an 11-point Likert-type scale (0 = *Not at all confident*, 10 = *Extremely confident*) and provided an explanation of their reasoning for their choice (in response to the free-response prompt: "Why did you choose this principle? Please describe what motivated you to make this decision").

Next, participants were asked whether they would repeat their choice in a hypothetical additional round. Each participant was randomly allocated to one of the four fields (ranging from least to most dense). In both conditions, participants were informed which field they would occupy in the hypothetical round. Participants then reported which principle they would choose for the hypothetical additional round.

Participants completed a questionnaire with Likert-type scales measuring risk preferences (0 = *Not at all willing to take risks*, 10 = *Very willing to take risks*) (1, 2), liberal-conservative political orientation (0 = *Strongly liberal*, 6 = *Strongly conservative*) (3), and left-right political orientation (0 = *Left*, 10 = *Right*) (4).

Participants next provided information on their age bracket (Table S6), gender, educational level (Table S7), income level (Table S8), handedness and video-game experience. Finally, the study asked participants to provide feedback on accessibility issues with the study and on the study in general.

Participants were debriefed about the study objectives and about deception in this study (e.g. that the other group members were bots, rather than human participants). The debrief acknowledged that participants might have foregone the highest possible benefit to themselves, in order to help other participants in this study. To avoid disadvantaging those participants, all participants were paid the highest possible bonus for the harvesting task (£1.50).

Study 3 followed the same stages as studies 1 and 2. However, rather than taking participants through the distribution process with written descriptions, the study ran an immersive harvesting game to create the group outcome distribution and simulate support from the AI assistant (Fig. S1). Study 3 contained minor changes to the instructions reflecting the longer study duration, higher base compensation, and the particular rules and controls for the immersive game (Figs. S10, S12, S13, and S14). To familiarize participants with the keyboard controls for the game, participants completed a one-minute tutorial before the actual harvesting round (Fig. S11).

While the comprehension test in studies 1 and 2 focused on matching each of the principles to corresponding outcome distributions, in study 3 the test required participants to match each of the principles to example videos of the AI assistant's harvesting behavior. Just before the test, participants watched several example videos showing the assistant following the principles. The assistant followed different paths in each video, so participants did not see the same exact behavior from the AI assistant twice. Participants watched at least one video for each of the principles, and could opt out in order not to watch a second video for each principle. Afterward, they watched two new videos of the AI assistant harvesting and matched them to the two principles (Figs. S15 and S16).

In the immersive game, the group outcome distribution shown after harvesting reflected the actual number of trees harvested by the participant, the AI assistant, and the other group members (Figs. S19 and S20).

Studies 4 and 5 tested boundary conditions for the effects of the VoI on decision making through small modifications to the basic experimental protocol. Study 4 informed participants that the other members of their group were not human participants, but computer-controlled bots. Participants were further instructed that the bots would complete the task (harvesting trees) just like they would. Unlike the other studies, we did not simulate waiting times throughout the experiment. Before selecting the decision maker to choose a principle, the study informed participants that bots could also be selected to act as the decision maker. The study reminded participants that the other members of their group were bots before they selected a principle.

Study 5 did not provide participants with written descriptions of the prioritarian and maximization principles. It instead referred to the principles as “Principle A” and “Principle B”, and relied on the visual depictions (i.e. bar charts) to communicate the effects of the principle on the distribution of outcomes for group members.

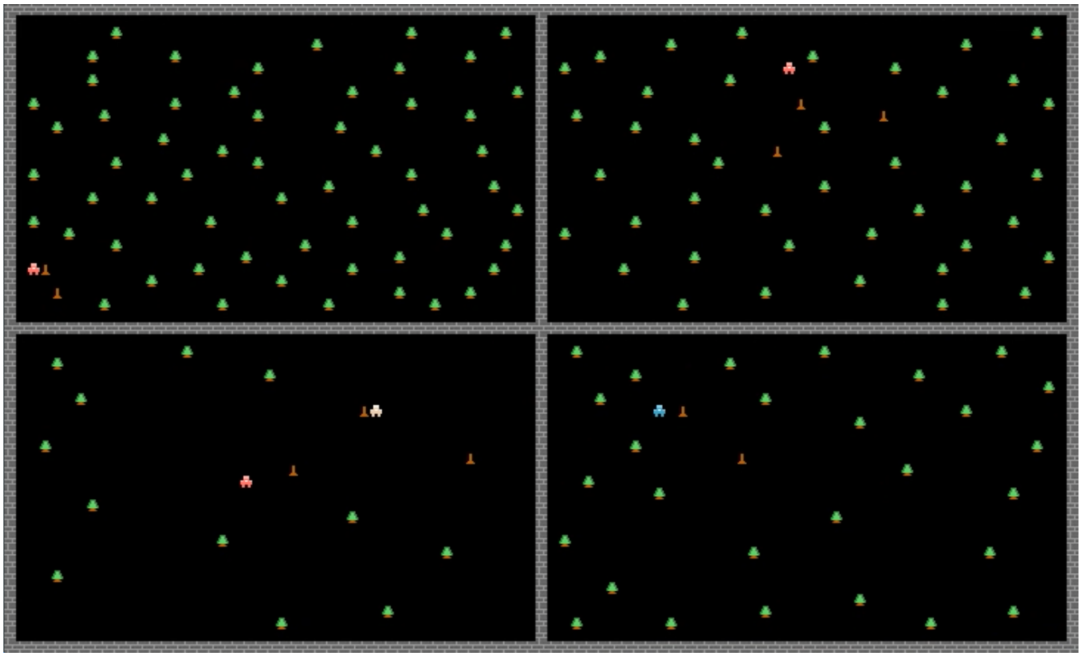

Fig. S1. The immersive harvesting game. The participant (blue figure) is assigned to the second most sparse field (lower right). The other group members (actually computer-controlled bots; red figures) were assigned to the other three fields. The AI assistant (beige figure) is currently contributing to the harvest in the sparsest field (lower left).

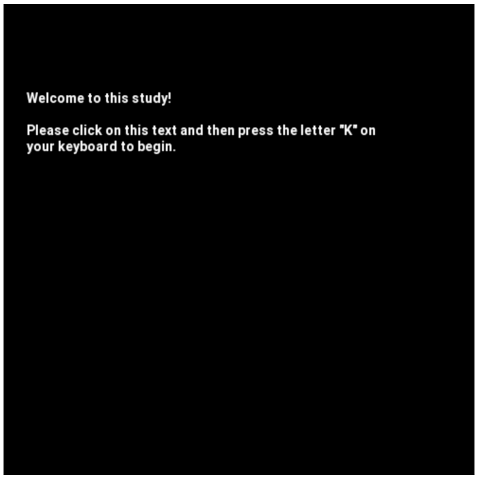

(a) Screen 1: Welcome participants to the experiment.

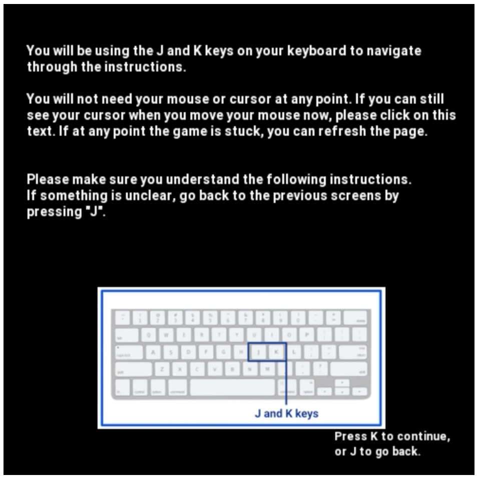

(b) Screen 2: Explain the keyboard controls.

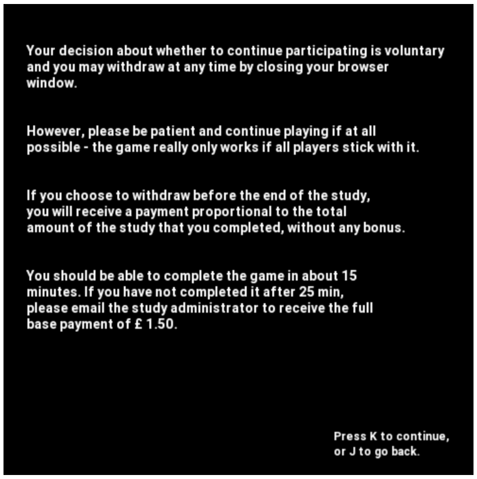

(c) Screen 3: Provide overview of the experiment logistics.

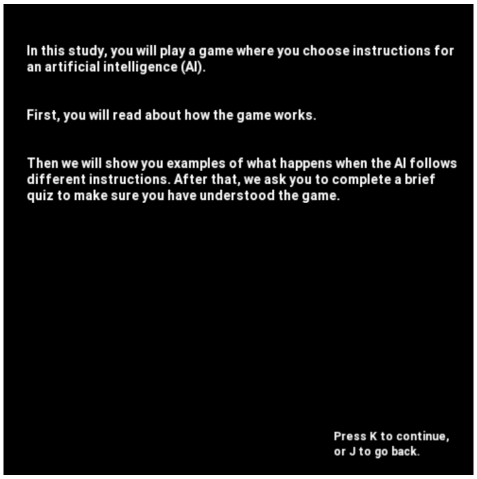

(d) Screen 4: Provide overview of how the study will unfold.

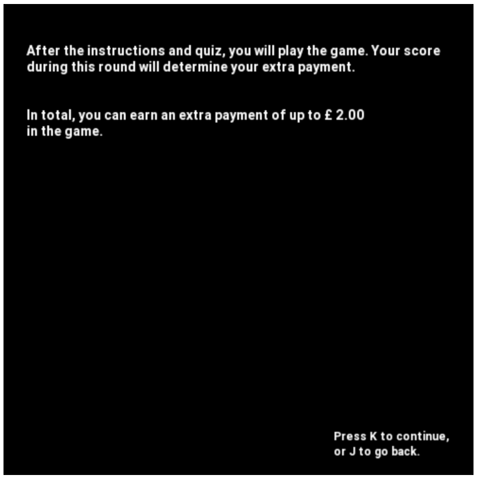

(e) Screen 5: Provide overview of payment.

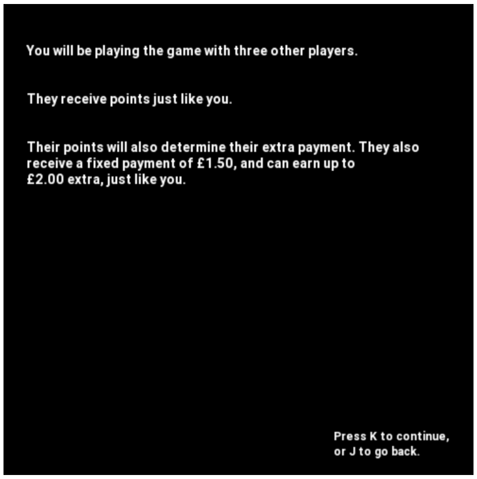

(f) Screen 6: Introduce the other group members.

Fig. S2. Screenshots of study instructions in studies 1 and 2.

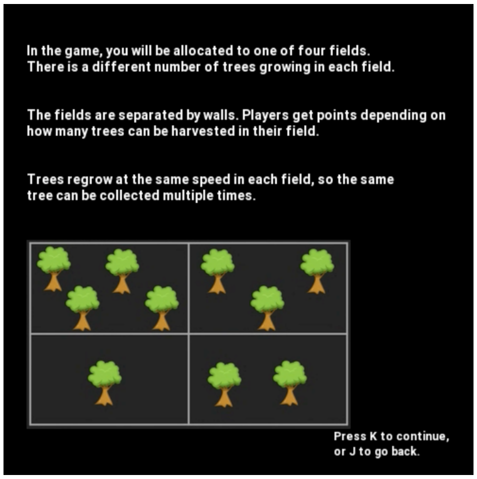

(a) Screen 7: Explain fields and tree harvesting.

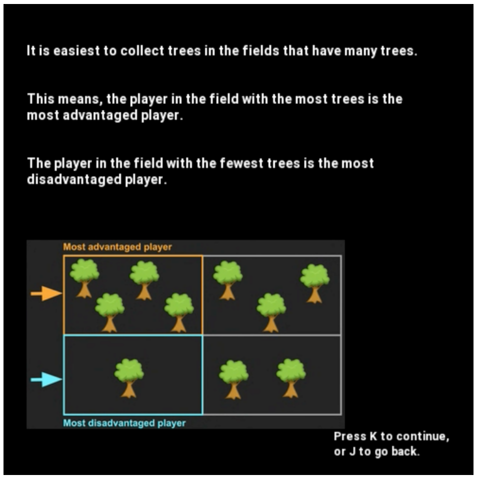

(b) Screen 8: Explain fields and tree harvesting.

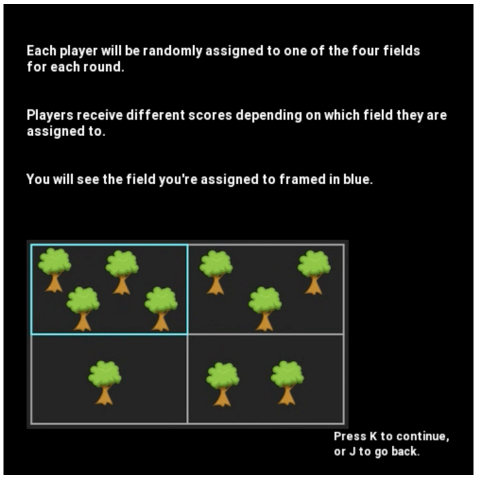

(c) Screen 9: Explain random assignment of group members to fields.

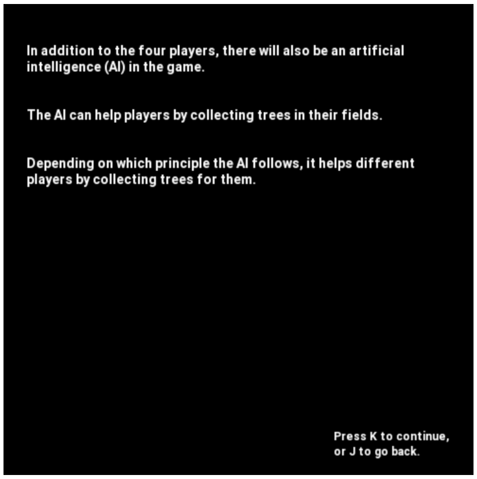

(d) Screen 10: Introduce the AI assistant.

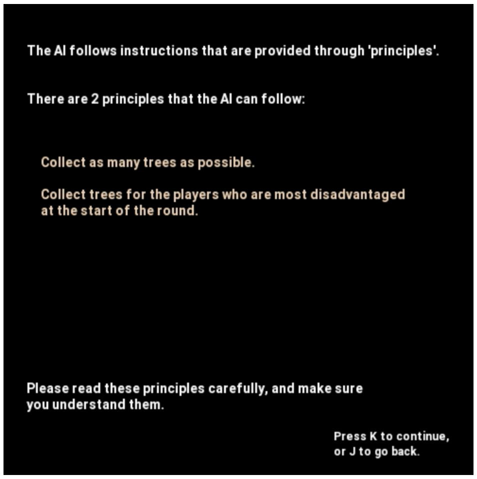

(e) Screen 11: Provide written descriptions of the principles that can guide the AI assistant.

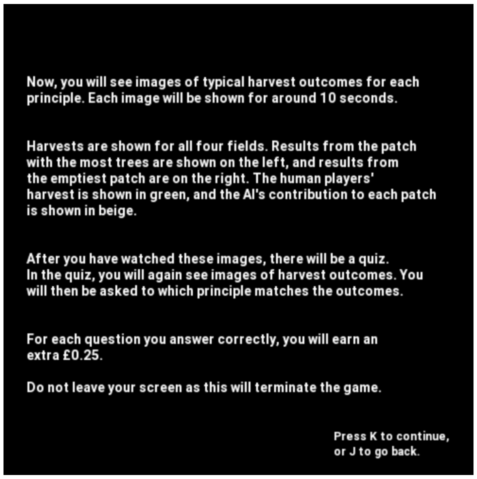

(f) Screen 12: Explain outcome distribution and comprehension test.

Fig. S3. Screenshots of instruction screens in studies 1 and 2.

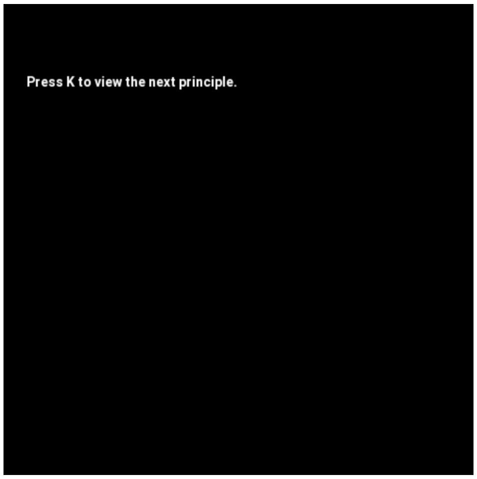

(a) Screen 13: Confirm participant is ready to view the first principle.

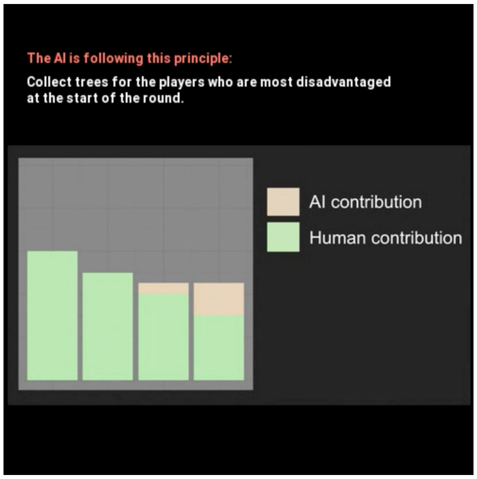

(b) Screen 14: Provide visual demonstration of a principle.

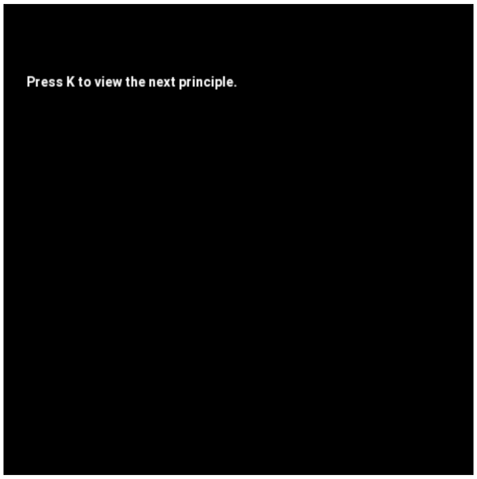

(c) Screen 15: Confirm participant is ready to view the next principle.

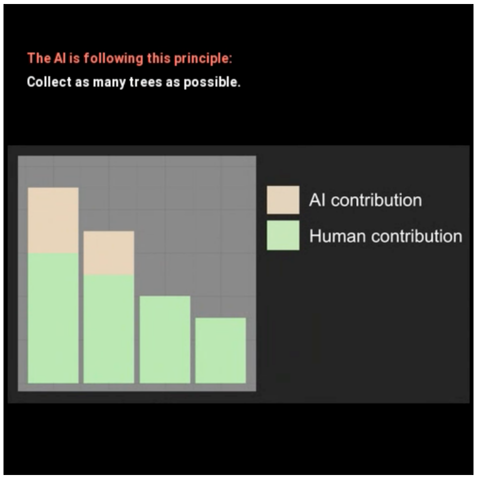

(d) Screen 16: Provide visual demonstration of a principle.

Fig. S4. Screenshots of instruction screens in studies 1 and 2.

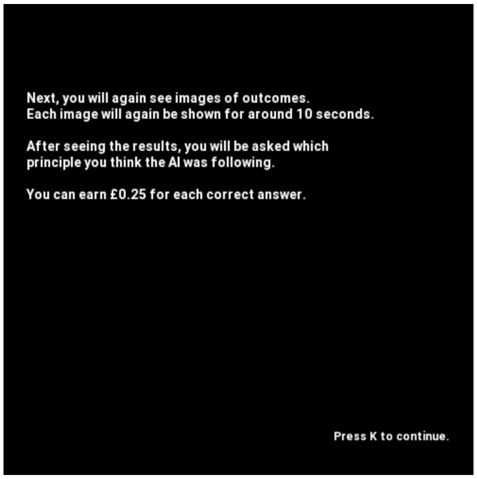

(a) Screen 17: Remind participant of rules for the comprehension test.

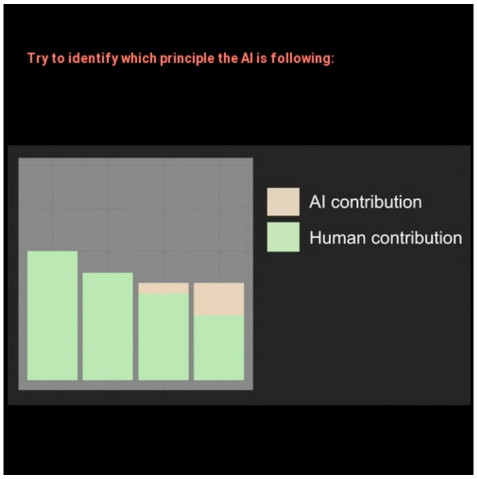

(b) Screen 18: Provide visual demonstration of a principle.

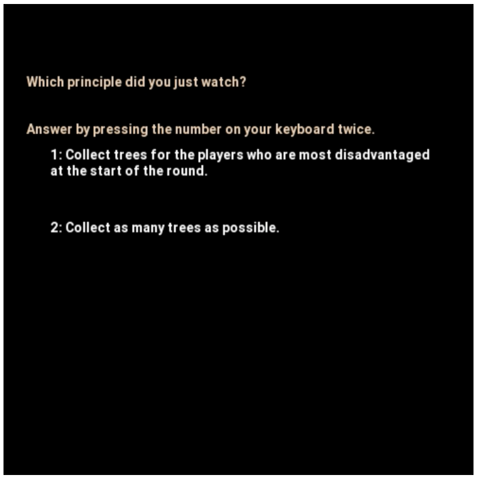

(c) Screen 19: Ask participant to identify which principle they observed.

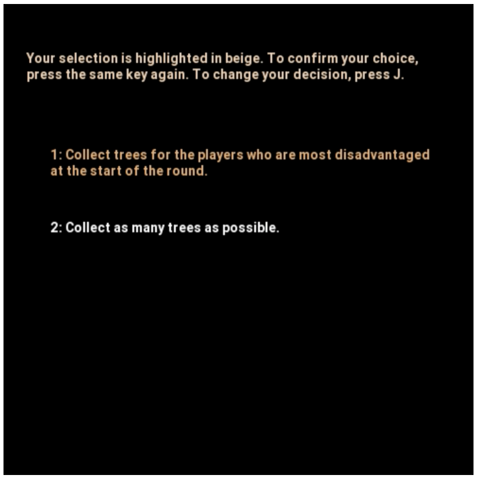

(d) Screen 20: Confirm participant's answer.

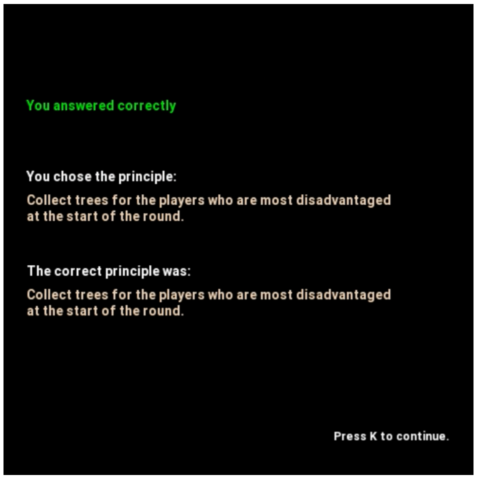

(e) Screen 21: Provide feedback on participant's answer.

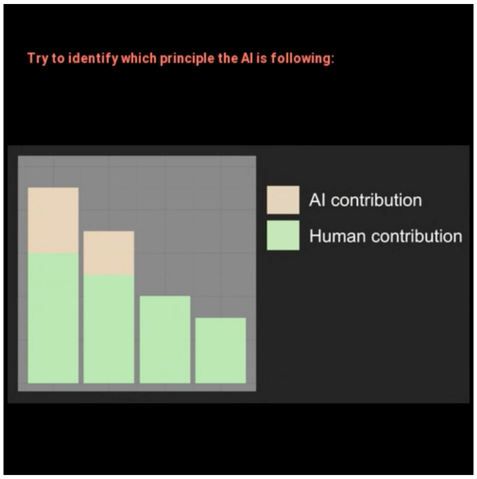

(f) Screen 22: Provide visual demonstration of a principle.

Fig. S5. Screenshots of the comprehension test in studies 1 and 2.

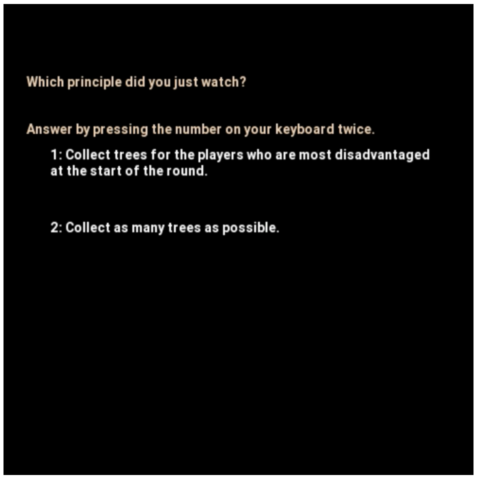

(a) Screen 23: Ask participant to identify which principle they observed.

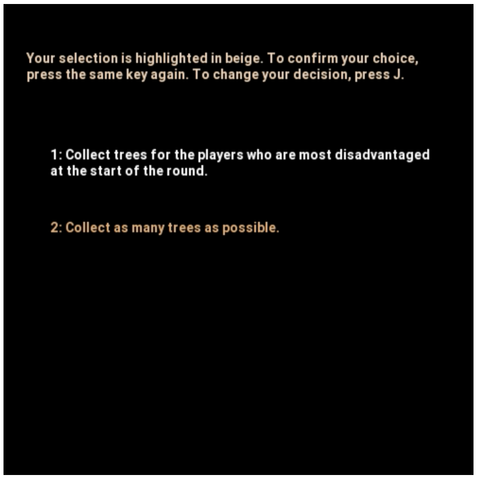

(b) Screen 24: Confirm participant's answer.

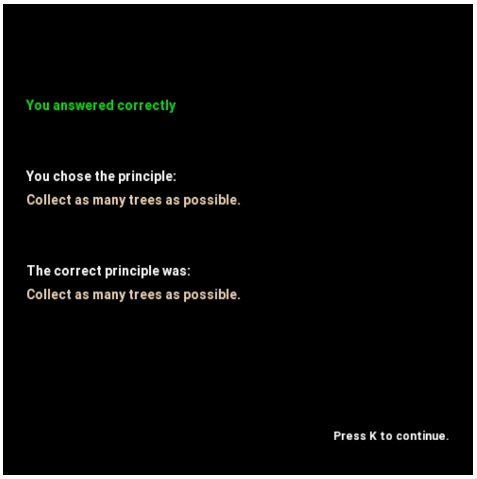

(c) Screen 25: Provide feedback on participant's answer.

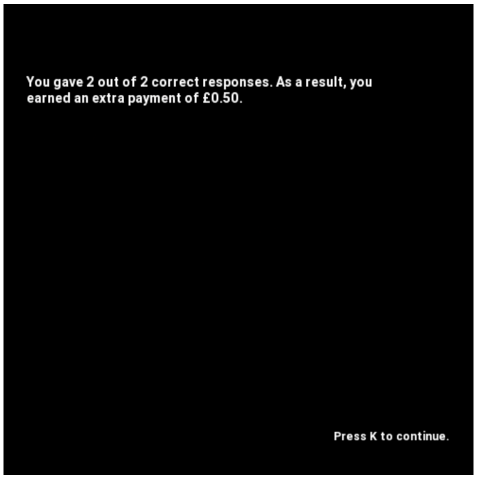

(d) Screen 26: Summarize test performance.

Fig. S6. Screenshots of the comprehension test in studies 1 and 2.

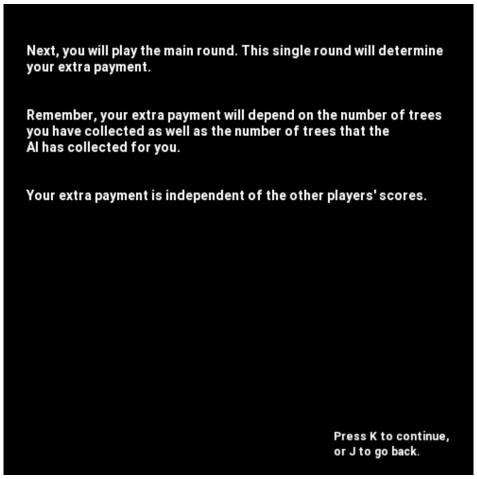

(a) Screen 27: Introduce the harvesting task.

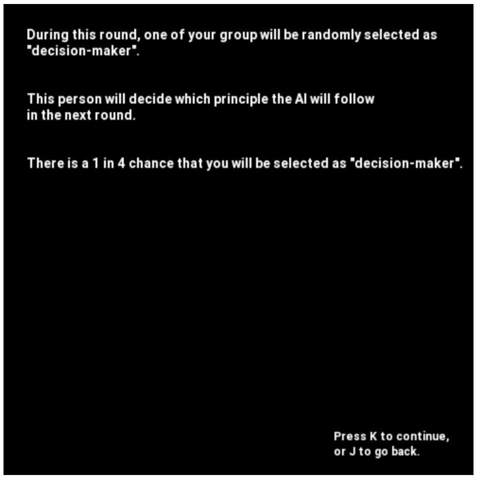

(b) Screen 28: Explain the principle selection process.

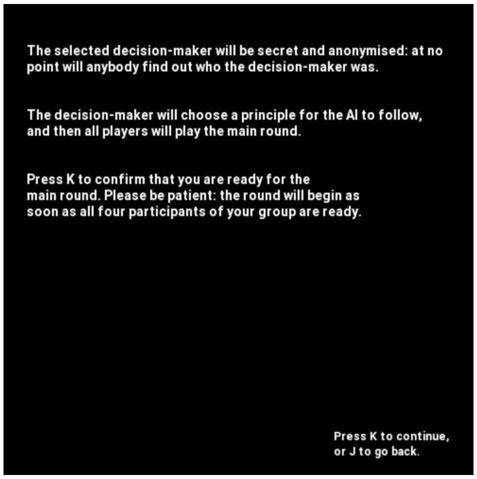

(c) Screen 29: Explain the principle selection process.

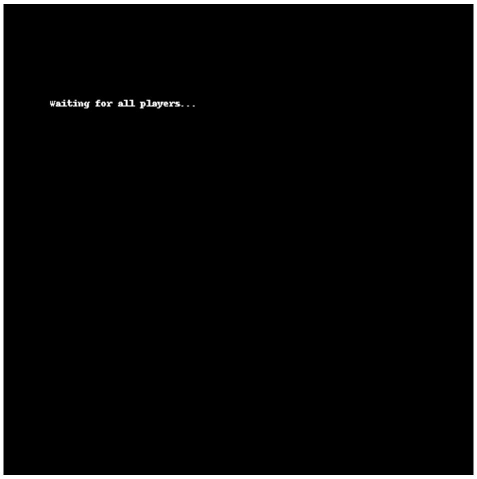

(d) Screen 30: Show waiting screen.

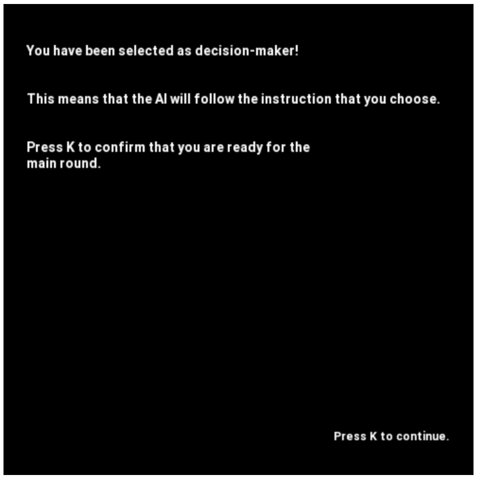

(e) Screen 31: Inform participant that they are the decision maker.

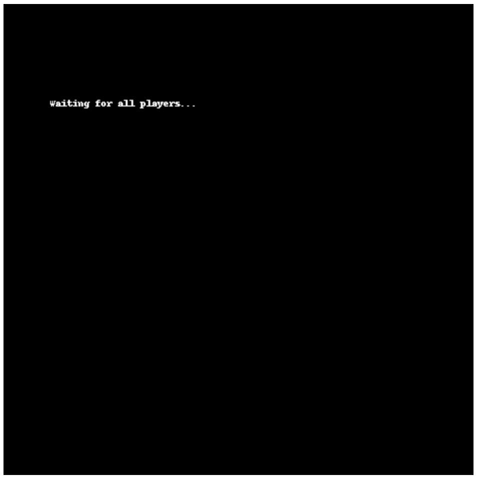

(f) Screen 32: Show waiting screen.

Fig. S7. Screenshots of instruction screens in studies 1 and 2.

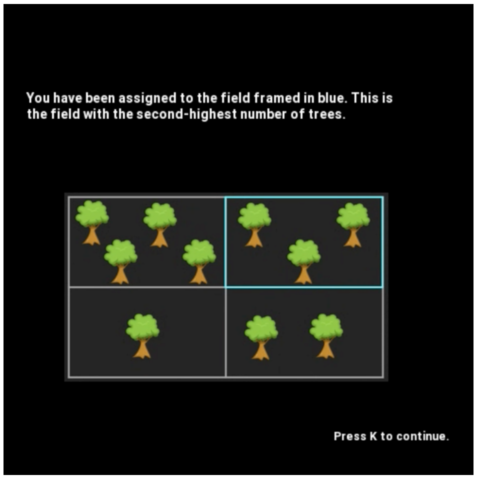

(a) Screen 33: Inform participant of their field (shown only in Control condition).

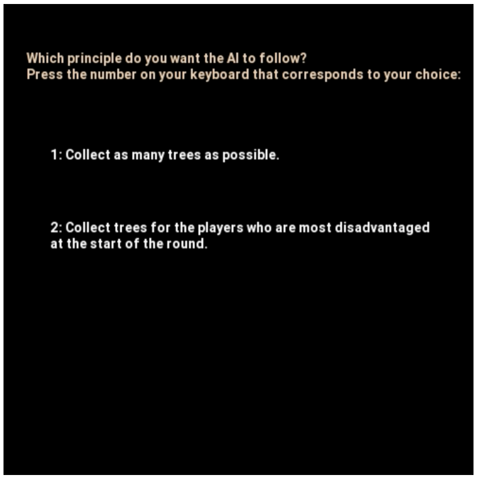

(b) Screen 34: Solicit principle choice from participant.

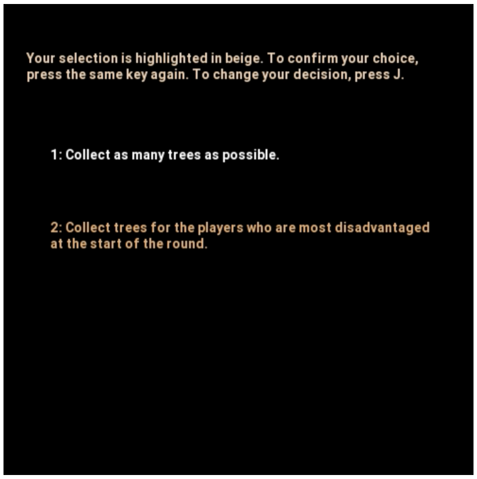

(c) Screen 35: Confirm principle choice from participant.

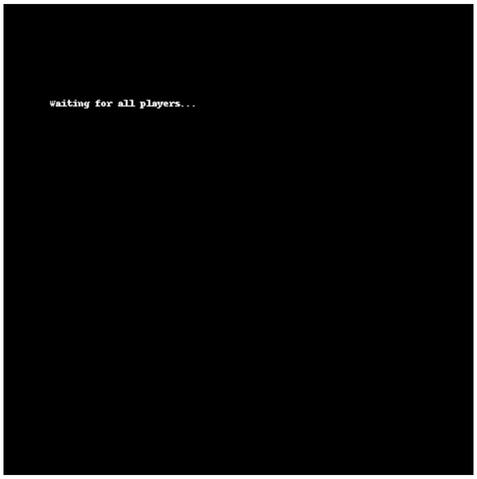

(d) Screen 36: Show waiting screen.

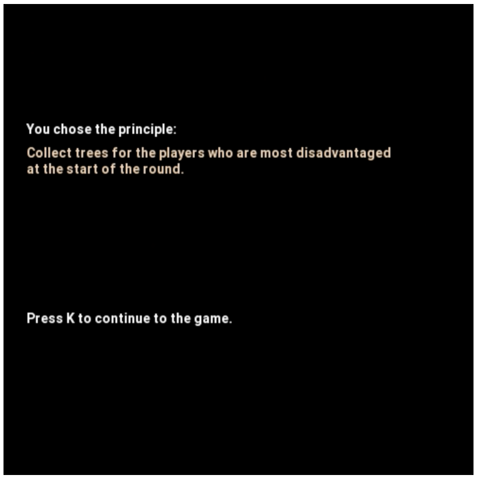

(e) Screen 37: Highlight principle choice for the group.

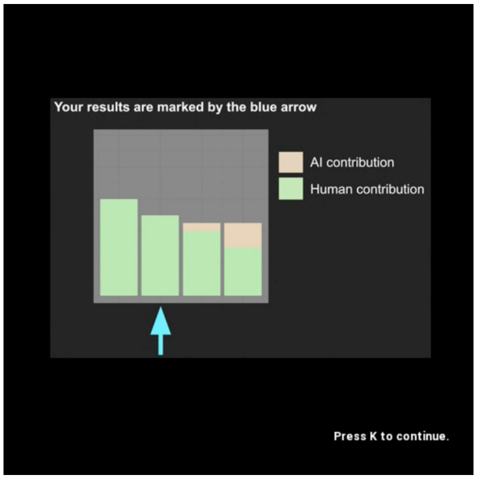

(f) Screen 38: Show outcome distribution for the group.

Fig. S8. Screenshots of the principle choice stage and harvesting task in studies 1 and 2.

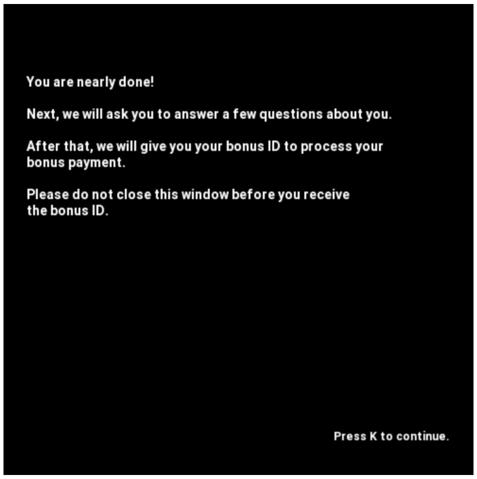

(a) Screen 39: Introduce questionnaire portion of the study.

Fig. S9. Screenshots of the post-task transition in studies 1 and 2.

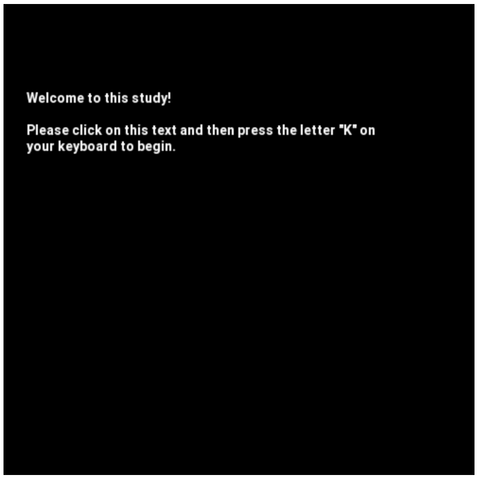

(a) Screen 1: Welcome participants to the experiment.

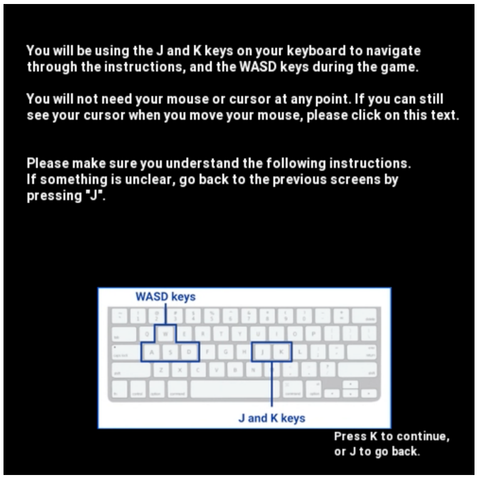

(b) Screen 2: Explain the keyboard controls.

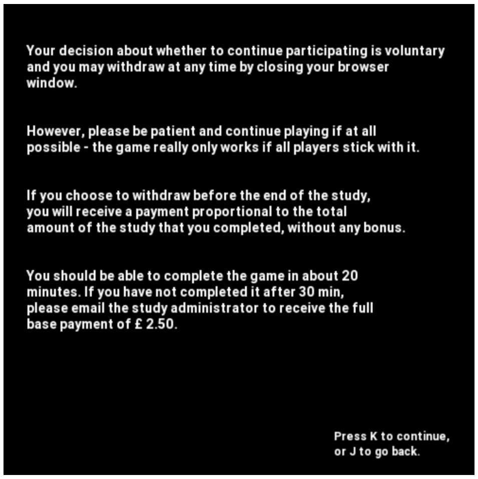

(c) Screen 3: Provide overview of the experiment logistics.

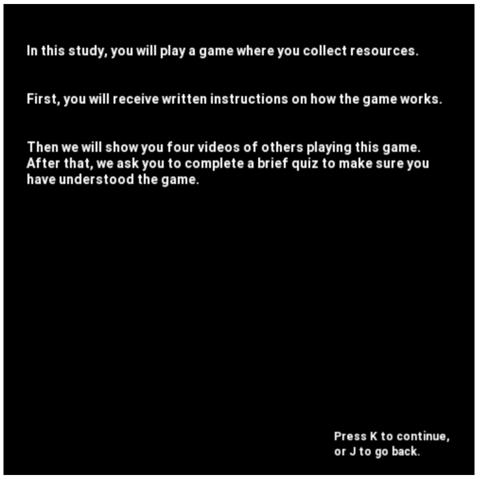

(d) Screen 4: Provide overview of how the study will unfold.

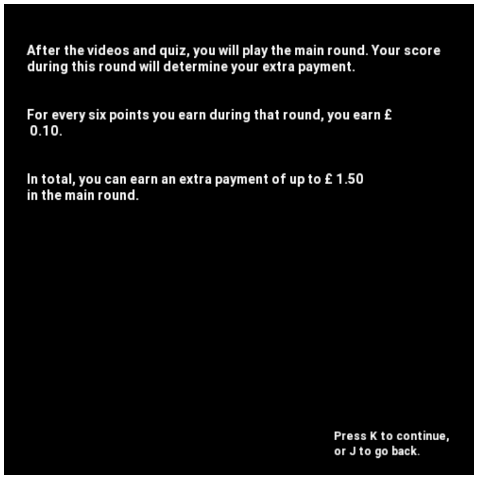

(e) Screen 5: Provide overview of how the study will unfold.

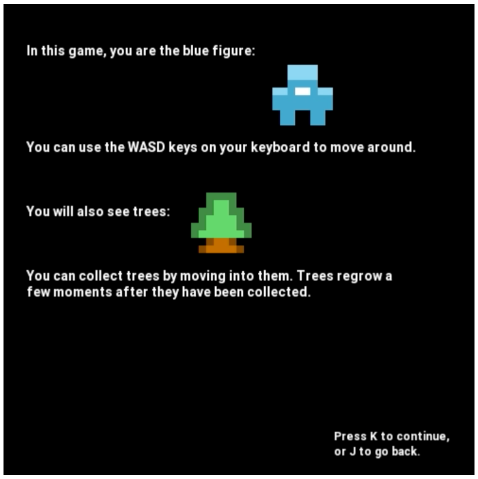

(f) Screen 6: Introduce the participant's avatar and tree harvesting.

Fig. S10. Screenshots of instruction screens in study 3.

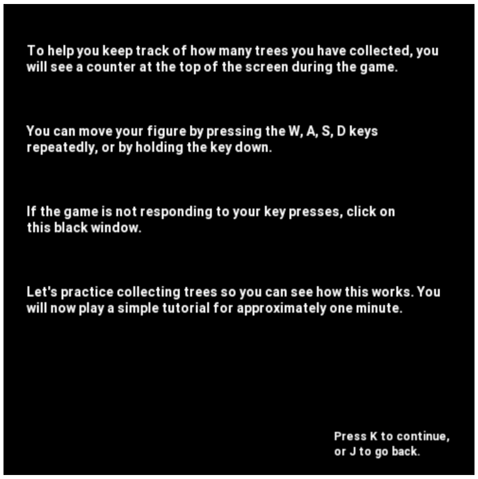

(a) Screen 7: Explain game controls and introduce tutorial.

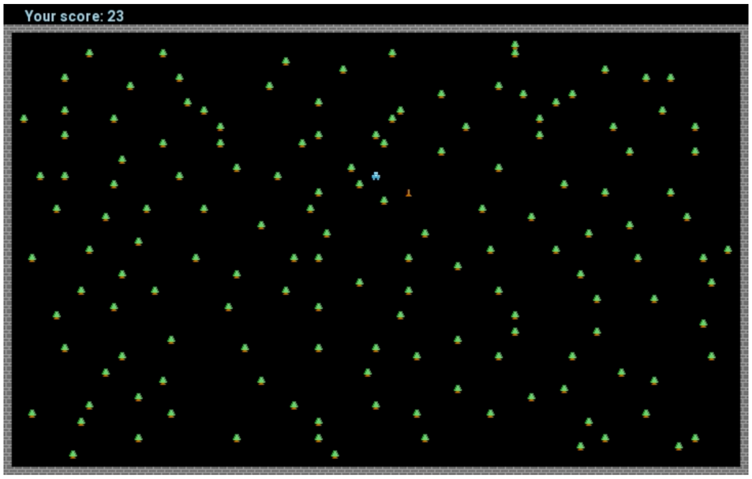

(b) Screen 8: Screenshot of harvesting tutorial.

Fig. S11. Screenshot of instruction screens and harvesting tutorial in study 3.

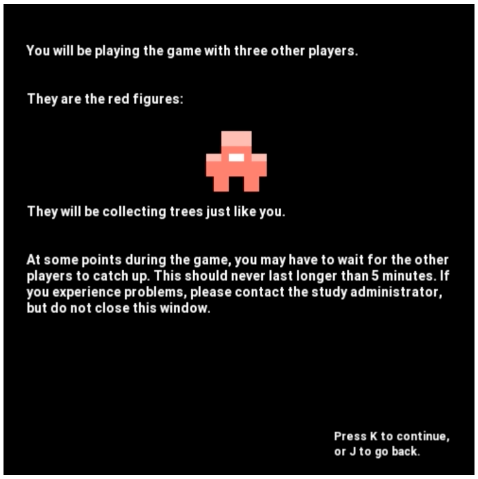

(a) Screen 9: Introduce the other group members.

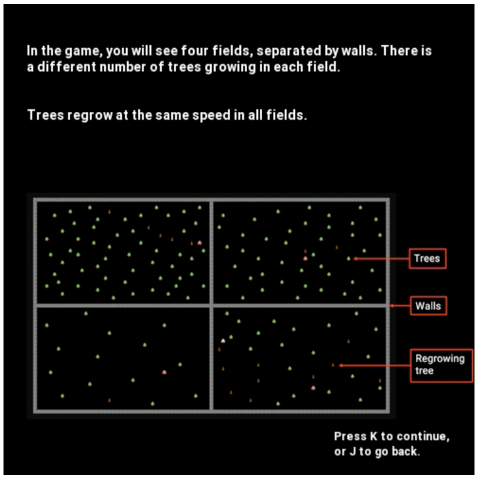

(b) Screen 10: Explain fields.

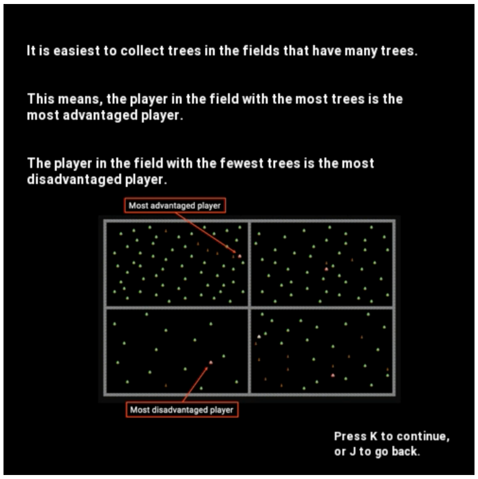

(c) Screen 11: Explain fields and tree harvesting.

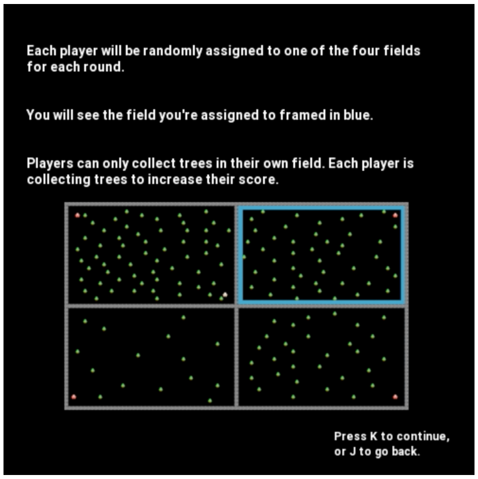

(d) Screen 12: Explain random assignment of group members to fields.

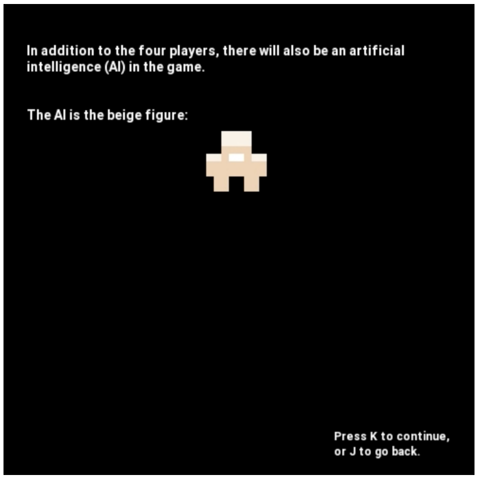

(e) Screen 13: Introduce the AI assistant.

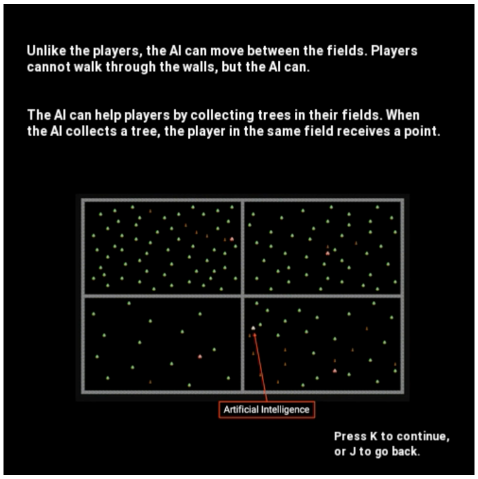

(f) Screen 14: Explain how the AI assistant helps harvest trees for group members.

Fig. S12. Screenshots of instruction screens in study 3.

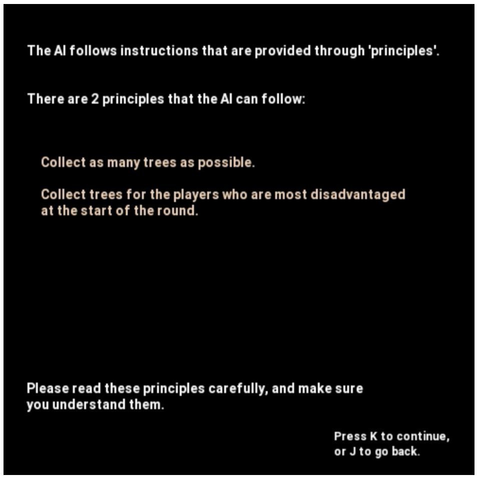

(a) Screen 15: Provide written descriptions of the principles that can guide the AI assistant.

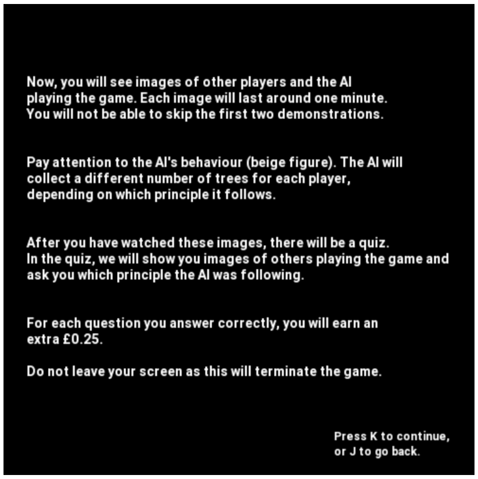

(b) Screen 16: Explain visual demonstrations and comprehension test.

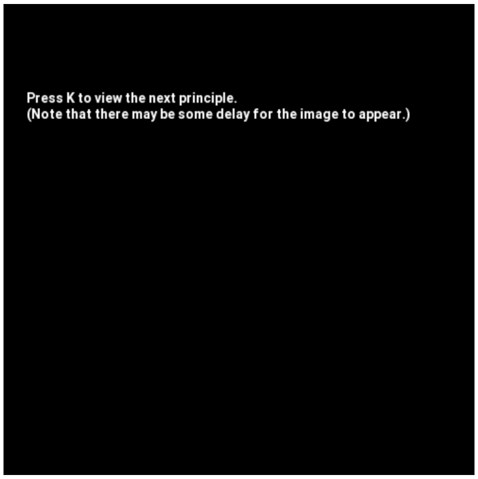

(c) Screen 17: Confirm participant is ready to view the first principle.

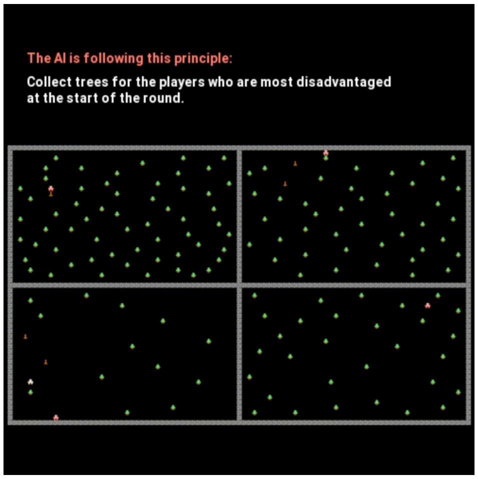

(d) Screen 18: Provide visual demonstration of a principle.

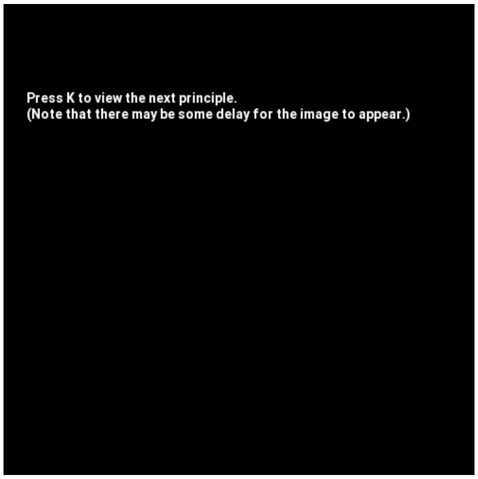

(e) Screen 19: Confirm participant is ready to view the next principle.

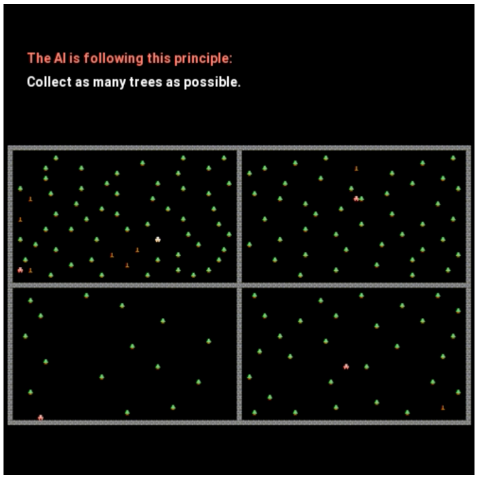

(f) Screen 20: Provide visual demonstration of a principle.

Fig. S13. Screenshots of instruction screens in study 3.

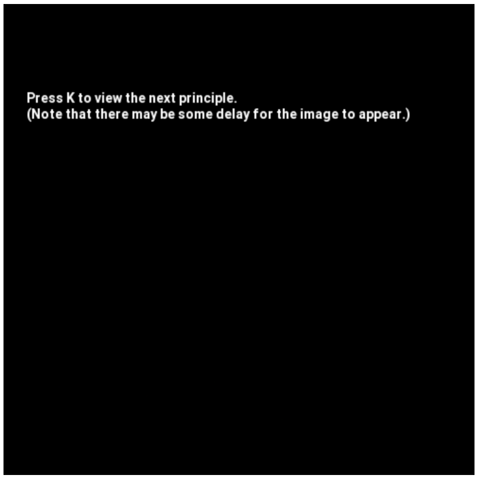

(a) Screen 21: Confirm participant is ready to view the next principle.

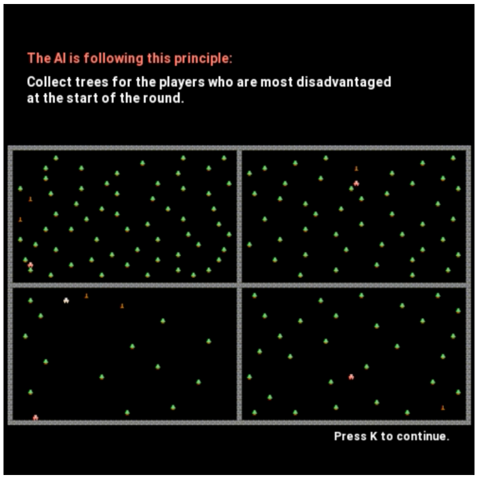

(b) Screen 22: Provide second visual demonstration of a principle.

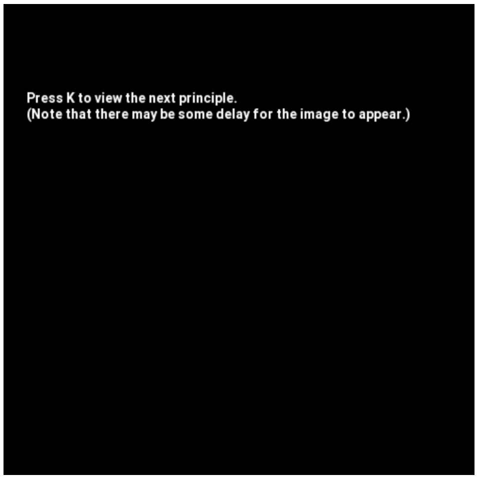

(c) Screen 23: Confirm participant is ready to view the next principle.

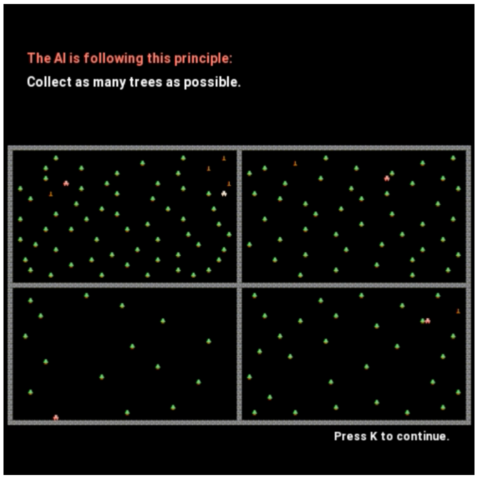

(d) Screen 24: Provide second visual demonstration of a principle.

Fig. S14. Screenshots of instruction screens in study 3.

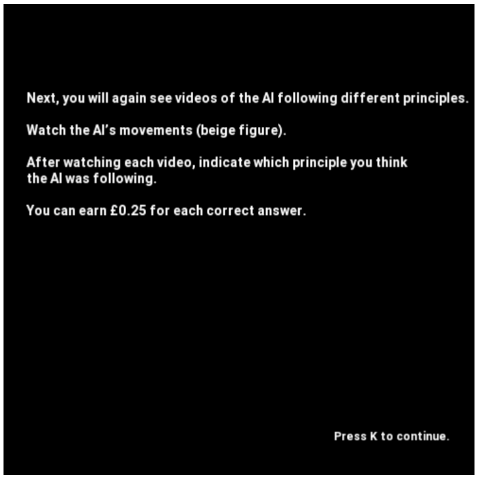

(a) Screen 25: Remind participant of rules for the comprehension test.

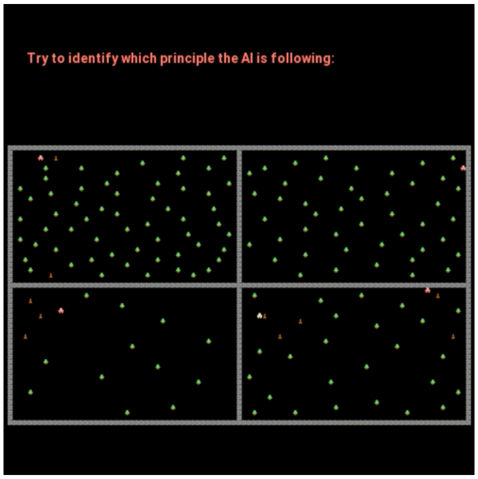

(b) Screen 26: Provide visual demonstration of a principle.

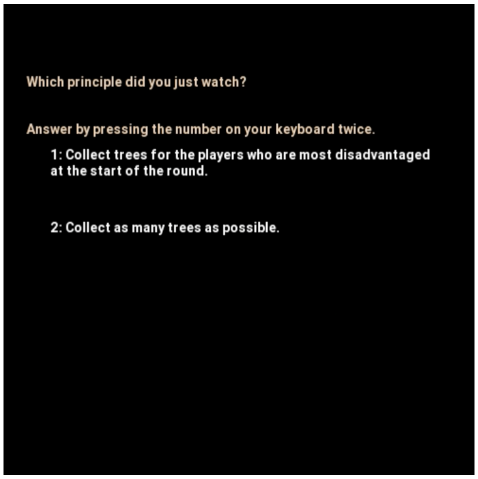

(c) Screen 27: Ask participant to identify which principle they observed.

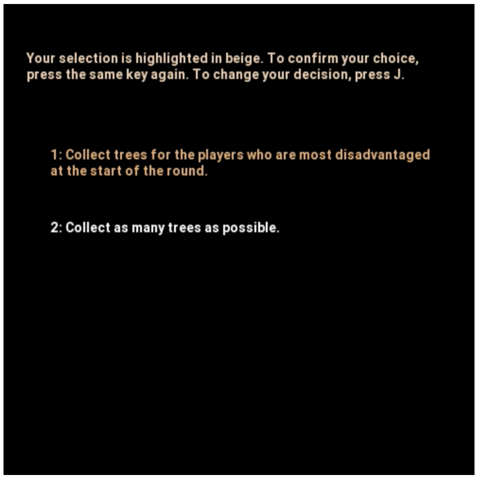

(d) Screen 28: Confirm participant's answer.

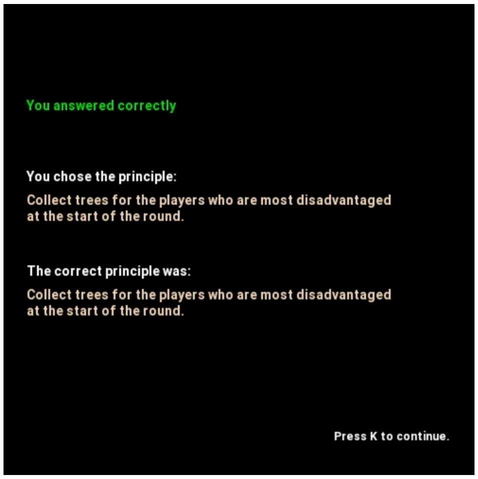

(e) Screen 29: Provide feedback on participant's answer.

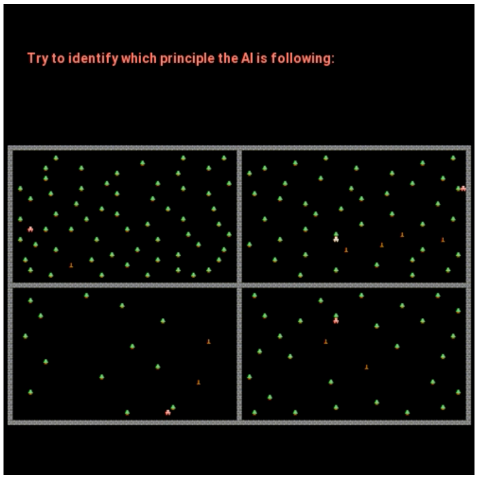

(f) Screen 30: Provide visual demonstration of a principle.

Fig. S15. Screenshots of comprehension test in study 3.

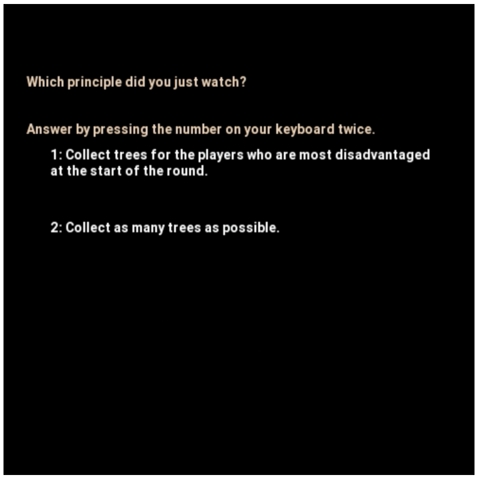

(a) Screen 31: Ask participant to identify which principle they observed.

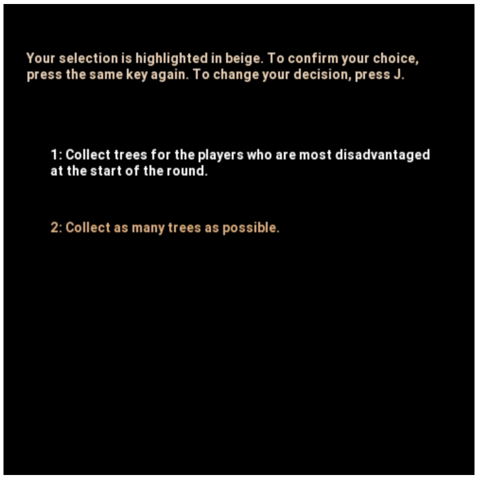

(b) Screen 32: Confirm participant's answer.

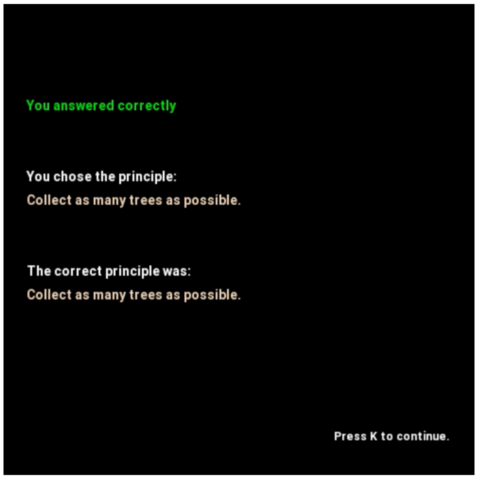

(c) Screen 33: Provide feedback on participant's answer.

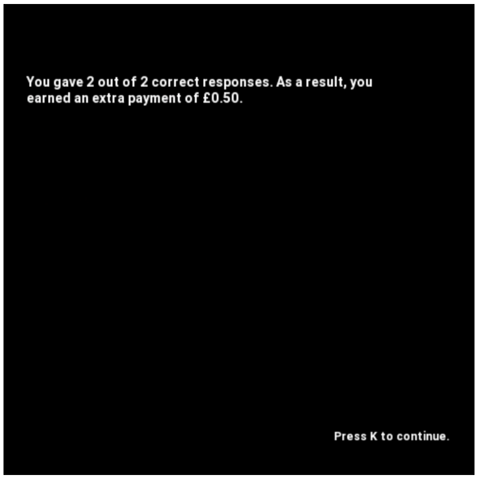

(d) Screen 34: Summarize test performance.

Fig. S16. Screenshots of comprehension test in study 3.

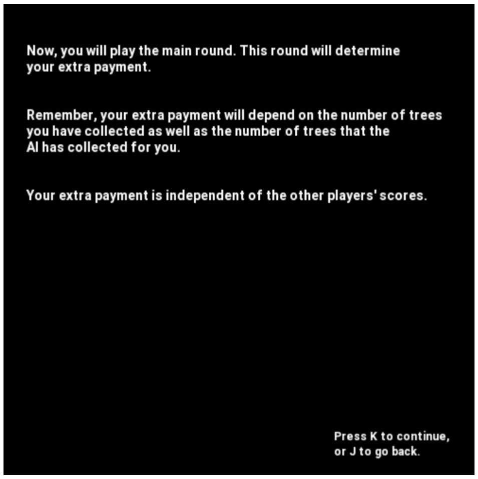

(a) Screen 35: Introduce the harvesting game.

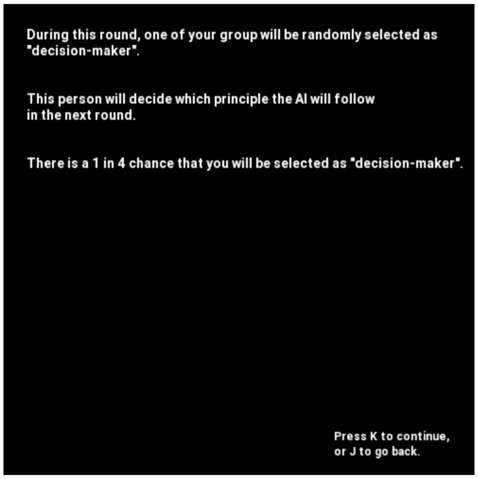

(b) Screen 36: Explain the principle selection process.

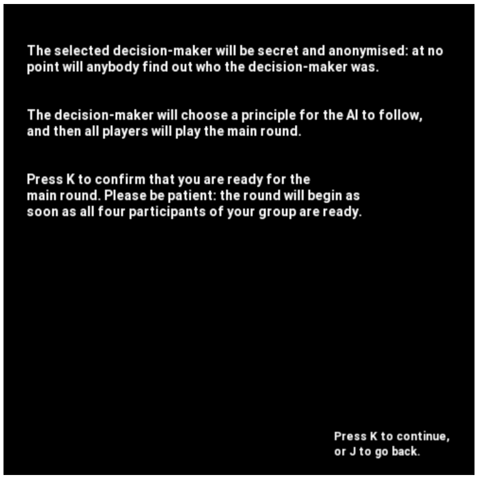

(c) Screen 37: Explain the principle selection process.

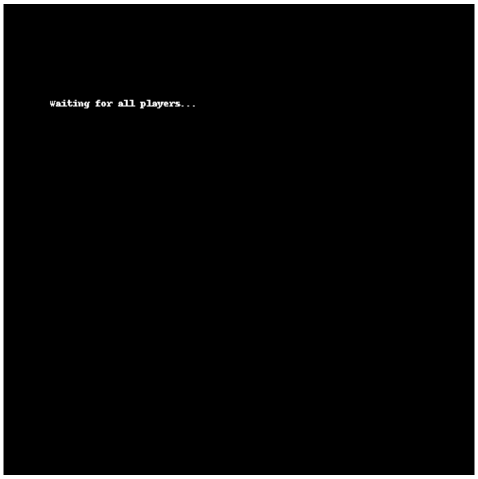

(d) Screen 38: Show waiting screen.

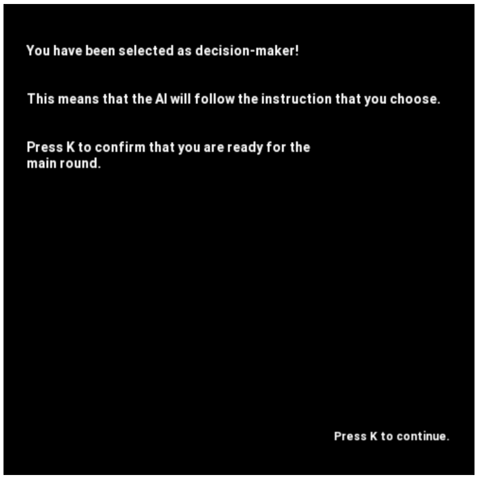

(e) Screen 39: Inform participant that they are the decision maker.

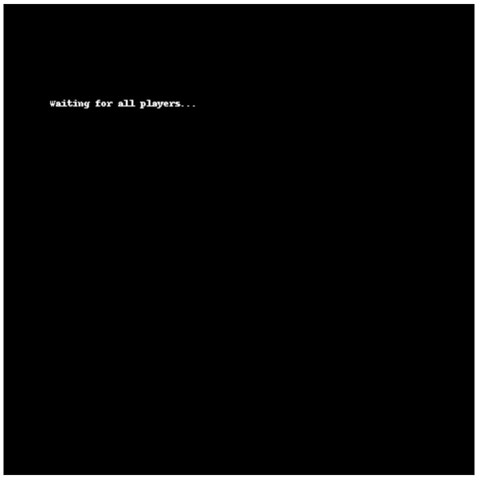

(f) Screen 40: Show waiting screen.

Fig. S17. Screenshots of instruction screens in study 3.

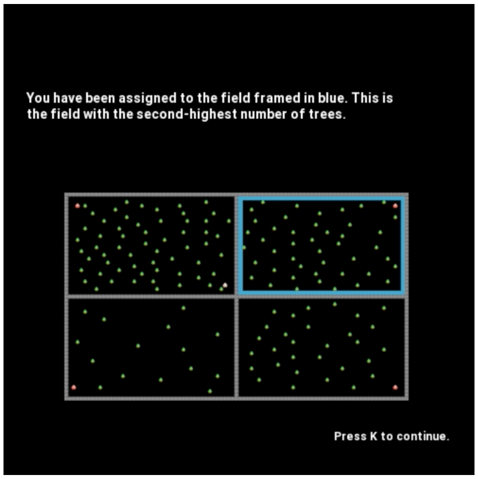

(a) Screen 41: Inform participant of their field (shown only in Control condition).

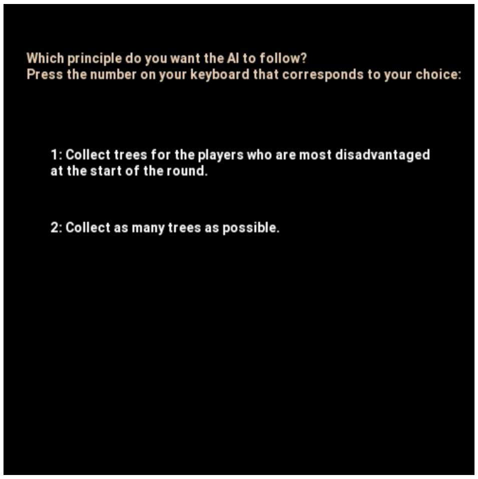

(b) Screen 42: Solicit principle choice from participant.

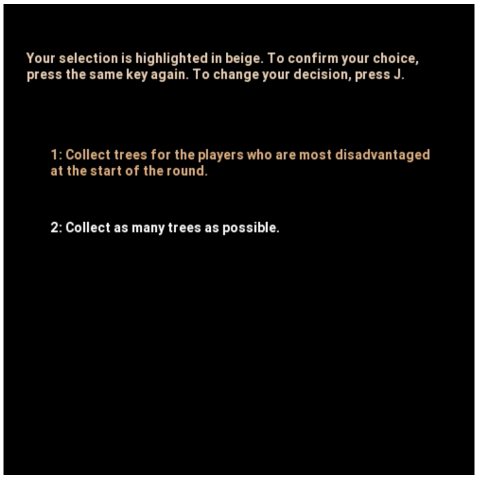

(c) Screen 43: Confirm principle choice from participant.

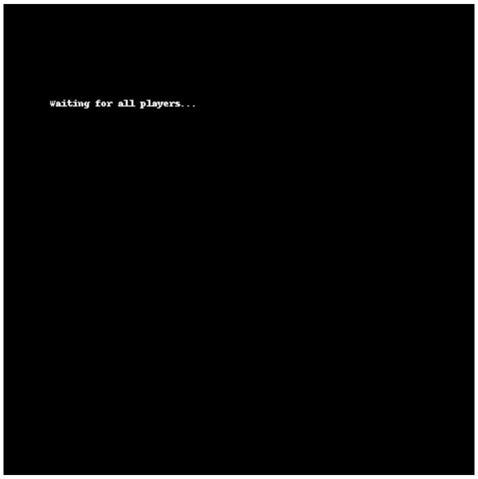

(d) Screen 44: Show waiting screen.

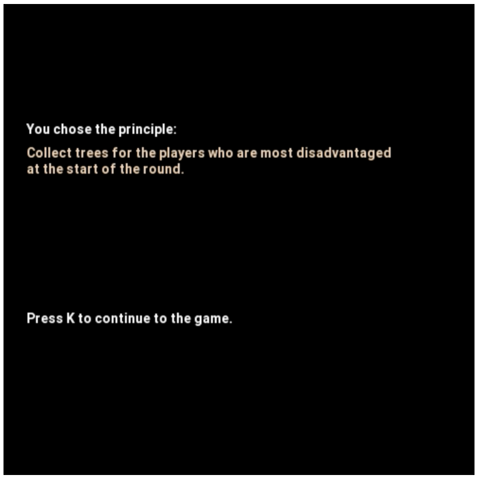

(e) Screen 45: Highlight principle choice for the group.

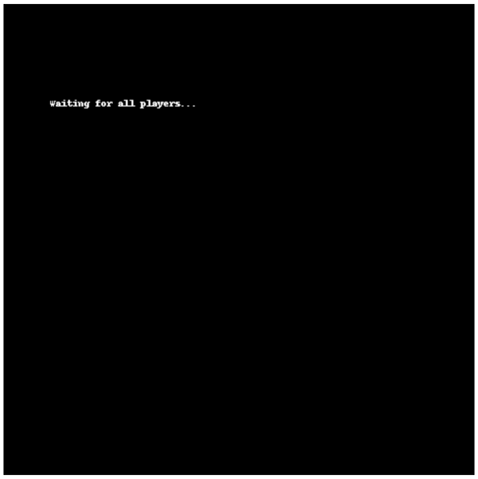

(f) Screen 46: Show waiting screen.

Fig. S18. Screenshots of principle choice stage in study 3.

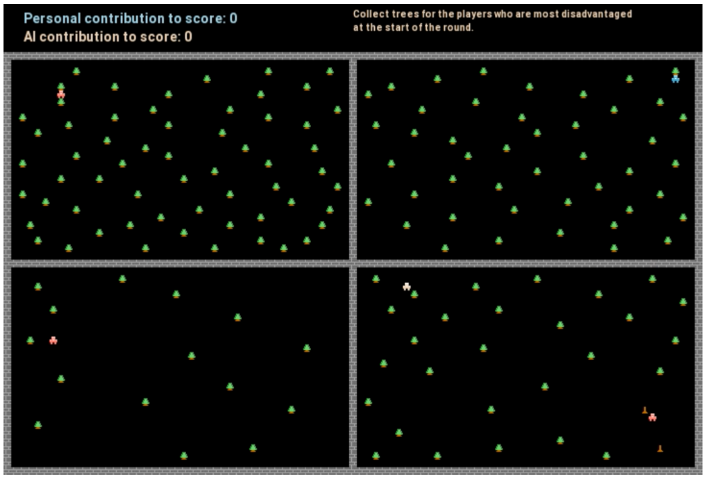

(a) Screen 47: Screenshot of harvesting game.

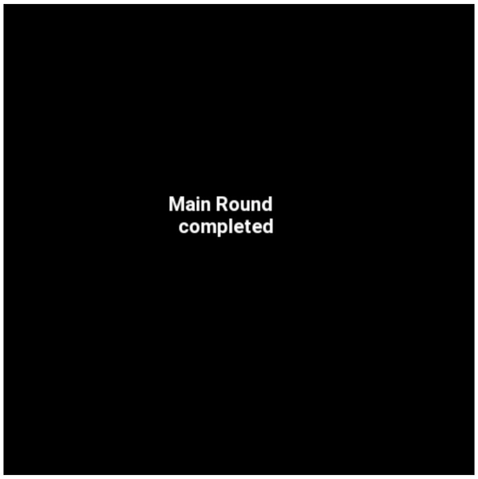

(b) Screen 48: Confirm that the group completed the harvesting game.

Fig. S19. Screenshots of harvesting game in study 3.

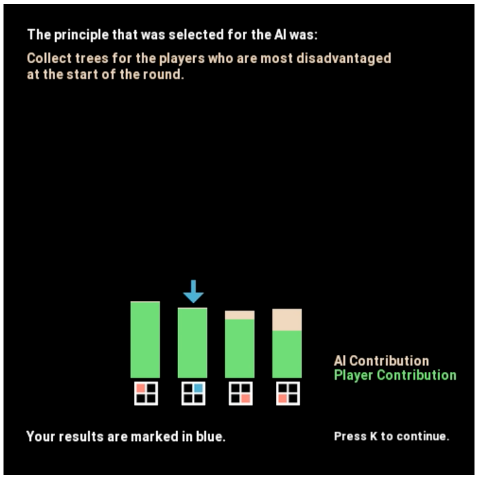

(a) Screen 49: Show outcome distribution for the group.

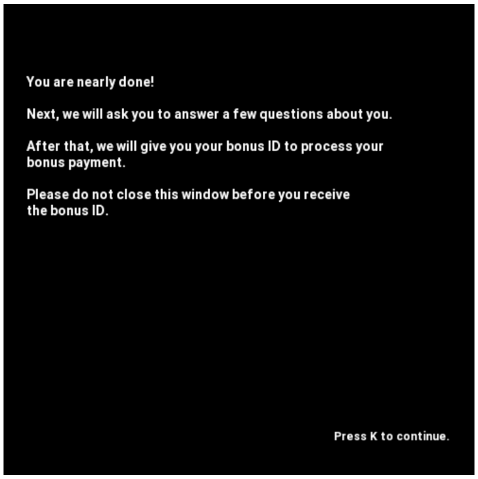

(b) Screen 50: Introduce questionnaire portion of the study.

Fig. S20. Screenshots of harvesting game and post-task transition in study 3.

Table S1. Results from logistic regressions analyzing the effect of the VoI on prioritarian choice. Cells present odds ratios representing the effect of the VoI condition (relative to the Control condition) on prioritarian choice.

	Study 1	Study 2	Study 3	Study 4	Study 5
Condition (0 = Control, 1 = VoI)	9.3*** [2.7, 37.6]	2.6*** [1.5, 4.5]	2.2* [1.2, 4.0]	4.5* [1.4, 16.8]	2.3 [0.6, 8.6]

95% confidence interval in brackets. * $p < 0.05$, ** $p < 0.01$, *** $p < 0.001$

Table S2. Results from logistic regressions analyzing the effect of the VoI on reflective endorsement. Cells present odds ratios representing the following effects on reflective endorsement: (1) the effect of the VoI condition (relative to the Control condition); (2) the effect of experiencing a motivation to change (relative to not experiencing such a motivation); and (3) the effect of the interaction of condition and experiencing a motivation to change.

	Study 1	Study 2	Study 3	Study 4	Study 5
(1) Condition (0 = Control, 1 = VoI)	0.6 [0.3, 1.3]	0.5** [0.3, 0.8]	0.9 [0.5, 1.7]	0.5 [0.2, 1.0]	0.7 [0.3, 1.6]
(2) Motivation to change (0 = Absent, 1 = Present)	0.1*** [0.0, 0.2]	0.1*** [0.0, 0.1]	0.2*** [0.1, 0.5]	0.1*** [0.0, 0.3]	0.1*** [0.0, 0.3]
(3) Condition : Motivation to change	8.6** [2.3, 34.0]	3.8*** [1.9, 7.7]	3.3* [1.1, 10.5]	2.3 [0.6, 8.6]	3.7 [1.0, 14.3]

95% confidence interval in brackets. * $p < 0.05$, ** $p < 0.01$, *** $p < 0.001$

Table S3. Reflective endorsement patterns observed in Study 1. Cells present the proportion of participants in a particular condition who chose a particular principle to guide the AI system and who either repeated or changed their principle choice for a hypothetical second round of the harvesting task.

Condition	Repeat	Change
Original principle choice: Prioritization		
Control	0.333	0.667
VoI	0.824	0.176
Original principle choice: Maximization		
Control	0.125	0.875
VoI	0.300	0.700

Table S4. Reflective endorsement patterns observed in Study 3. Cells present the proportion of participants in a particular condition who chose a particular principle to guide the AI system and who either repeated or changed their principle choice for a hypothetical second round of the harvesting task.

Condition	Repeat	Change
Original principle choice: Prioritization		
Control	0.467	0.533
VoI	0.684	0.316
Original principle choice: Maximization		
Control	0.300	0.700
VoI	0.545	0.455

Table S5. Results from simple logistic regressions predicting prioritarian choice behind the VoI. Cells present odds ratios representing (non-standardized) effects of the predictors on prioritarian choice among participants in the VoI condition.

	Study 1	Study 2	Study 3	Study 4	Study 5
Attitudes to risk	0.82* (0.065)	0.86*** (0.039)	0.92 (0.012)	0.76*** (0.089)	1.0 (0.001)
Political orientation (liberal-conservative)	0.79 (0.025)	0.84* (0.022)	0.88 (0.012)	0.67** (0.096)	0.87 (0.014)
Political orientation (left-right)	0.91 (0.010)	0.88* (0.024)	0.90* (0.017)	0.78* (0.067)	1.01 (0.000)
Fairness-based reasoning	28.0*** (0.512)	46.7*** (0.597)	22.9*** (0.484)	12.8*** (0.298)	16.1*** (0.391)

Nagelkerke's R^2 in parentheses. * $p < 0.05$, ** $p < 0.01$, *** $p < 0.001$

Table S6. Distribution of participant age groups across all studies (pre-exclusion).

Age group	N
18-24	309
25-34	732
35-44	676
45-54	411
55-64	264
65+	116

Table S7. Distribution of participants' educational levels across all studies (pre-exclusion).

Educational level	N
High school	646
No graduation	130
University degree (Bachelor or Master)	1,452
Graduate degree (PhD)	123
Other	145
Prefer not to say	12

Table S8. Distribution of participant income levels across all studies (pre-exclusion).

Annual income	N
Below £10,000	438
£10,000 to £30,000	1,143
More than £30,000	806
Prefer not to say	121

References

1. T Dohmen, et al., Individual risk attitudes: Measurement, determinants, and behavioral consequences. *J. Eur. Econ. Assoc.* **9**, 522–550 (2011).
2. F Aguiar, A Becker, L Miller, Whose impartiality? an experimental study of veiled stakeholders, involved spectators and detached observers. *Econ. & Philos.* **29**, 155–174 (2013).
3. J Graham, J Haidt, BA Nosek, Liberals and conservatives rely on different sets of moral foundations. *J. Pers. Soc. Psychol.* **96**, 1029 (2009).
4. M Kroh, Measuring left–right political orientation: The choice of response format. *Public Opin. Q.* **71**, 204–220 (2007).
